# Spatiotemporal tracking of gold nanorods after intranasal administration for brain targeting

**DOI:** 10.1016/j.jconrel.2023.04.022

**Published:** 2023-05

**Authors:** Shunping Han, Julie Tzu-Wen Wang, Emine Yavuz, Alaa Zam, Nadia Rouatbi, Rifka Nurul Utami, Revadee Liam-Or, Alexander Griffiths, Wayne Dickson, Jane Sosabowski, Khuloud T. Al-Jamal

**Affiliations:** a Institute of Pharmaceutical Science, Faculty of Life Sciences & Medicine, King's College London, Franklin-Wilkins Building, 150 Stamford Street, London SE1 9NH, United Kingdom; b London Metallomics Facility, King's College London, Franklin-Wilkins Building, 150 Stamford Street, London SE1 9NH, United Kingdom; c Department of Physics, King's College London, Strand, London WC2R 2LS, United Kingdom; d Centre for Molecular Oncology, Barts Cancer Institute, Queen Mary University of London, Charterhouse Square, London EC1M 6BQ, United Kingdom; e Advanced Technology Research and Application Center, Selcuk University, Aleaddin Keykubat Yerleskesi, Akademi Mah. Yeni Istanbul Cad. No: 355/C, Selcuklu, Konya, Turkey; f London Centre for Nanotechnology, King's College London, Strand, London WC2R 2LS, United Kingdom

**Keywords:** Gold nanorods, Intranasal administration, Biodistribution, Brain region-specific uptake, Multi-modal imaging, Glioma

## Abstract

Intranasal administration is becoming increasingly more attractive as a fast delivery route to the brain for therapeutics circumventing the blood-brain barrier (BBB). Gold nanorods (AuNRs) demonstrate unique optical and biological properties compared to other gold nanostructures due to their high aspect ratio. In this study, we investigated for the first time the brain region-specific distribution of AuNRs and their potential as a drug delivery platform for central nervous system (CNS) therapy following intranasal administration to mice using a battery of analytical and imaging techniques. AuNRs were functionalized with a fluorescent dye (Cyanine5, Cy5) or a metal chelator (diethylenetriaminepentaacetic dianhydride, DTPA anhydride) to complex with Indium-111 *via* a PEG spacer for optical and nuclear imaging, respectively. Direct quantification of gold was achieved by inductively coupled plasma mass spectrometry. Rapid AuNRs uptake in mice brains was observed within 10 min following intranasal administration which gradually reduced over time. This was confirmed by the 3 imaging/analytical techniques. Autoradiography of sagittal brain sections suggested entry to the brain *via* the olfactory bulb followed by diffusion to other brain regions within 1 h of administration. The presence of AuNR in glioblastoma (GBM) tumors following intranasal administration was also proven which opens doors for AuNRs applications, as nose-to-brain drug delivery carriers, for treatment of a range of CNS diseases.

## Highlights

• Multi-modal imaging techniques were applied to understand the spatiotemporal biodistribution of gold nanorods.
• Gold nanorods demonstrated rapid brain uptake following intranasal administration.
• Gold nanorods first reached olfactory bulbs within minutes and gradually distributed to other brain regions with time.

## Graphical abstract

**Figure:**
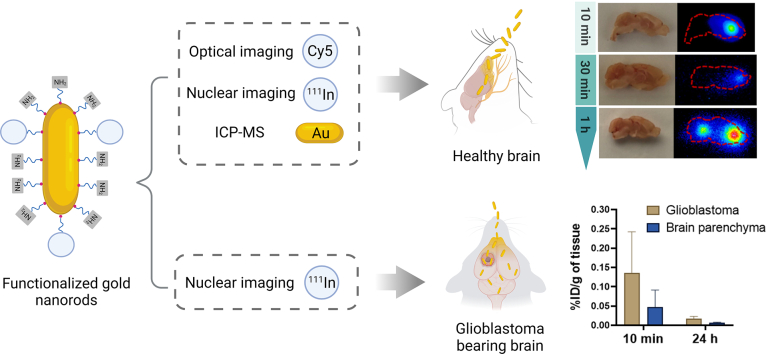

## 1 Introduction

Delivery of therapeutics for the treatment of central nervous system (CNS) diseases is often challenged by the presence of the blood-brain barrier (BBB). In general, only drugs with low molecular weights (under a threshold of 400–600 Da) and high lipid solubility can cross the BBB owing to the unique brain capillary endothelial cell structures with tight junctions and efflux transporters [[1], [2], [3]]. These features limit most drugs from reaching the brain at therapeutic levels. For decades, great efforts have been undertaken to achieve sufficient brain accumulation of the therapeutic agents including using invasive techniques (*e.g.*, Gliadel® wafers, intrathecal injection, and convection-enhanced delivery) [4,5], disruption of BBB (*e.g.*, osmotic disruption, ultrasound disruption, and magnetic disruption) [2] and targeting BBB receptors (*e.g.*, transferrin receptors and folate receptors) [2,4]. However, these strategies are either intrusive or lack specific targeting to disease sites, resulting in adverse effects.

Intranasal administration is an alternative route for delivering therapeutic agents to the brain. This non-invasive approach allows therapeutics to enter the brain through the olfactory and trigeminal nerve pathways, circumventing the BBB [6]. The benefits of this administration route also include the avoidance of first pass metabolism by the liver, reduced drug accumulation at non-targeted tissues, and rapid onset of actions. For these reasons, intranasal delivery has been trialled for a wide range of therapeutic agents such as proteins, peptides, and small molecules for CNS disease treatment [2,7]. For example, TNX-2900, an intranasal potentiated form of oxytocin, was approved by the U.S. Food and Drug Administration (FDA) as a treatment for Prader-Willi syndrome. Midazolam is an FDA approved nasal spray to treat seizures. In addition to the free drugs, lipid [8], polymer [9,10], chitosan [11] and inorganic material-based [12,13] nanoplatforms have also demonstrated promising effects for CNS disorder treatment after intranasal administration in preclinical studies.

Gold nanoparticles (AuNPs) and nanocrystals constitute the most advanced form of gold nanostructures that reached clinical trials for CNS applications [14,15]. The first-in-human trial to determine the safety of nucleic acid labelled gold nanoparticles in treating patients with recurrent glioblastoma multiforme or gliosarcoma after intravenous injection has been completed recently with 0% of mortality and 87.5% of patients unaffected by serious adverse events (ClinicalTrials.gov Identifier: NCT03020017). The assessment of oral gold nanocrystals' safety and efficacy for relapsing-remitting multiple sclerosis treatment is now in Phase 2 clinal trials (ClinicalTrials.gov Identifier: NCT03536559 and NCT03993171). Gold nanorods (AuNRs) constitute another form of gold nanostructures being increasingly investigated biologically due to their anisotropic structure, tuneable surface plasmon and excellent biocompatibility profiles [16]. In the past decades, AuNRs have been investigated for bioimaging [[17], [18], [19]], photothermal therapeutics [20] and drug delivery [21] applications for CNS diseases through systemic exposure mainly *via* the intravenous route. Published reports have shown that intratumorally or intravenously administered AuNRs combined with photothermal therapy can suppress the growth of glioblastoma tumors [20,22,23]. In addition, intravenous amyloid-β targeted inhibitory peptides [21] and quercetin [24] loaded onto AuNRs achieved therapeutic advantages in the neurodegenerative disease landscape. Only a few studies have been reported on intranasal delivery of gold nanostructures. They have mainly focused on using gold nanoparticles. Exploiting the intranasal route for AuNRs delivery remains an explored area.

To track gold nanostructures *in vitro* and *in vivo*, a number of imaging and quantification techniques have been employed such as magnetic resonance (MR) imaging [25,26], optical imaging [27], nuclear imaging [28,29] and inductively coupled plasma mass spectrometry (ICP-MS) [30,31]. Each of these techniques comes with its own set of benefits and limitations. For example, MR imaging can provide tomographic information on live biological specimens with high spatiotemporal resolution (down to 50–250 μm) but lacks intrinsic contrast which makes definitive detection of desired tissue difficult [25,32]. Optical imaging is highly desirable in clinical and preclinical studies due to its rapid screening and cost-effectiveness. However, background autofluorescence and limited light penetration into deep tissues make it difficult to quantitively analyze gold nanostructures [32,33]. ICP-MS is considered the ‘gold standard’ method for gold content quantification in tissues, however, it is labour intensive without offering spatial information. Therefore, multi-modal imaging techniques are required for robust qualitative and quantitative analyses of AuNR biodistribution profiles.

In this study, we built on our in-house expertise in multi-modal imaging to investigate the spatiotemporal distribution of AuNRs after intranasal administration. To achieve this, AuNRs were functionalized with Cyanine5 (Cy5), a fluorescent probe suitable for optical imaging, or diethylenetriaminepentaacetic dianhydride (DTPA dianhydride), a metal chelator, to enable nuclear imaging. Whole body distribution with a focus on healthy and glioblastoma bearing brains was comprehensively conducted using optical imaging, ICP-MS, single-photon emission computed tomography (SPECT)/computed tomography (CT) imaging, gamma counting and autoradiography. The results obtained provide evidence of AuNRs access to the brain *via* the nasal route which are highly relevant for further development of AuNRs as drug delivery carriers for brain imaging and therapy *via* the nose-to-brain route.

## 2 Materials and methods

### 2.1 Materials

Gold(III) chloride trihydrate (HAuCl_4_ · 3H_2_O), cetyltrimethylammonium bromide (CTAB), sodium borohydride (NaBH_4_), l-ascorbic acid (Vitamin C), diethylenetriaminepentaacetic dianhydride (DTPA anhydride), Ninhydrin reagent (2% solution), sodium carboxymethyl cellulose (CMC, Mw = ∼ 700,000) and Tween® 20 were purchased from Sigma-Aldrich (UK). Silver nitrate (AgNO_3_) was supplied by VWR International (UK). NH_2_-PEG-SH (amine-PEG_3500_-thiol, average Mw = 3500) was purchased from JenKem Technology (USA). Sulfo-cyanine5 NHS ester was purchased from Lumiprobe (USA). Nitric acid (HNO_3_, 68%, *w*/w) and hydrochloric acid (HCl, 37%, w/w) with trace metal grades for ICP-MS measurements were obtained from Thermo Fisher Scientific (UK). Indium-111 chloride was purchased from Curium (The Netherlands). Isoflurane (IsoFlo®) for anaesthesia was purchased from Zoetis (UK). Dialysis bags (SnakeSkin Dialysis Tubing, MWCO = 3500) were purchased from Thermo Fisher Scientific (UK). PD-10 columns and NAP™-5 columns were purchased from GE Healthcare Life Sciences (UK). Thin-layer chromatography (TLC) silica gel on aluminium sheets was purchased from Merck Millipore (UK). Instant TLC papers (iTLC-SG) were purchased from Agilent Technologies (UK). Other chemicals, not specified here, were all analytical grades purchased from standard chemical suppliers and used without further purification.

### 2.2 Synthesis of AuNRs

Gold nanorods were synthesized by the seed-mediated growth method as described previously with modifications [34,35]. Firstly, CTAB solution (5 mL, 0.2 M) was mixed with HAuCl_4_ · 3H_2_O (5 mL, 0.0005 M). Then ice-cold NaBH_4_ (600 μL, 0.01 M) was added to the above mixture, which rapidly turned into a brownish-yellow color. The solution was vigorously stirred for 2 min and kept in a water bath at 28 °C for 2 h and was used as seed solution.

For growth solution preparation, HAuCl_4_·3H_2_O (45 mL, 0.001 M) was mixed with AgNO_3_ (3.6 mL, 0.004 M) and CTAB (45 mL, 0.1 M). After mixing the solution a few times by inversion, vitamin C (0.72 mL, 0.1 M) and HCl (1.44 mL, 1 M) were added to the solution. Finally, the seed solution (360 μL) was added to the prepared growth solution. The whole mixture was kept at 28 °C overnight to obtain AuNRs.

After synthesis, the mixture was purified by a combinational purification method in which the mixture was firstly dialyzed (dialysis bag, MWCO = 3500) against deionized water (1500 mL) overnight with the external dialysis buffer exchanged once. Afterwards, the solution was split in two halves and transferred into two 50 mL centrifuge tubes and centrifuged (Eppendorf, Centrifuge 5810 R, UK) at 10,000 rpm for 15 min at room temperature (RT). The pellets were resuspended into deionized water (30 mL) and washed one more time before collection.

### 2.3 Synthesis of Cy5-AuNR_PEG-NH2_

#### 2.3.1 Cy5-PEG-SH synthesis

Sulfo-cyanine5 NHS ester was dissolved in dimethyl sulfoxide (DMSO) to obtain a concentration of 5 mM. NH_2_-PEG-SH (450 μL, 550 μM in 0.1 M sodium bicarbonate buffer (pH 8.2)) was mixed with sulfo-cyanine5 NHS ester (50 μL, 5000 μM in DMSO) at 1:1 molar ratio. After being reacted for 3 h at RT in the dark, the mixture was applied to a NAP™-5 column and eluted with deionized water. A total of 30 fractions of 120 μL each were collected. Afterwards, each fraction was diluted 100-fold with deionized water for fluorescence intensity measurement. In addition, 2 μL of each fraction was spotted on thin-layer chromatography (TLC) silica gel supported by aluminium sheet. The color was developed under iodine vapour to detect PEG fragments. Only fractions which were Cy5 (blue color) and PEG fragment (brown color) positive were collected. The final concentration of Cy5-PEG-SH was measured in a plate reader (BMG Labtech, UK) against a 0–5 μM Cy5 standard curve. Cy5-PEG-SH linkers were synthesized freshly before use.

#### 2.3.2 Cy5-AuNR_PEG-NH2_ synthesis

AuNR stock solution (1 mL, 50 nM) was centrifuged (10,000 rpm, 15 min, RT) to collect the pellet. A mixture (1 mL) containing Cy5-PEG-SH (200 μM) and NH_2_-PEG-SH (500 μM) was added to AuNR pellet fraction (30 μL) and then reacted for 24 h in the dark. As a negative control group, the mixture (1 mL) was mixed with deionized water (30 μL) and reacted under the same condition. At the end of the reaction, the mixtures were centrifuged (10,000 rpm, 15 min, RT). The concentration of Cy5 in the supernatant was diluted 100-fold with deionized water before being measured in the plate reader. The conjugated Cy5 on AuNRs was calculated by measuring Cy5 amount in the supernatant of the experimental group which was subtracted from the negative control group. The optical properties of Cy5-AuNR_PEG-NH2_ before and after functionalization were characterized using UV–vis–NIR spectra. The Cy5-AuNR_PEG-NH2_ pellets were resuspended into 0.1% Tween® 20 and washed once by centrifugation before use.

### 2.4 Synthesis of DTPA-AuNR_PEG-NH2_

#### 2.4.1 DTPA-PEG-SH synthesis

The reaction time and molar ratio between DTPA anhydride and NH_2_-PEG-SH were optimized to synthesize DTPA-PEG-SH. In brief, NH_2_-PEG-SH (2.5 mM final concentration) was mixed with DTPA anhydride (5, 2.5, 1.25 or 0.625 mM final concentration) in 500 μL DMSO. After being reacted for 4 h and 24 h, respectively, the substitution of amine groups in NH_2_-PEG-SH was determined by Ninhydrin assay (Supplementary Information).

An optimized NH_2_-PEG-SH and DTPA anhydride molar ratio of 1:1 was used for large-scale synthesis to obtain DTPA-PEG-SH linkers. Briefly, DTPA anhydride (3.6 mg) was dissolved in DMSO (1 mL) and then added to NH_2_-PEG-SH powder (35 mg). The mixture was stirred for 4 h. At the end of the reaction, the mixture was diluted 5-fold with deionized water before being applied to a PD-10 column and eluted with deionized water. A total of 30 fractions of 400 μL each was collected. PEG presence in each fraction was determined by TLC as described above. Only fractions positive for PEG were collected. To obtain a high purity of DTPA-PEG-SH for radiolabelling, DTPA-PEG-SH linkers were purified by the PD-10 column for another three rounds: two rounds were eluted with 0.9% NaCl and the last round was eluted with deionized water. The concentrations of PEG and DTPA fragments in the final products were quantified by the published iodine solution-based assay [36] and Gd^3+^-Xylenol Orange assay [37], respectively, with modifications (Supplementary Information). The resulting products after freeze-drying were stored at −20 °C until use.

#### 2.4.2 DTPA-AuNR_PEG-NH2_ synthesis

DTPA-PEG-SH (100 μL, 2.5 mM) and NH_2_-PEG-SH (100 μL, 2.5 mM) were added to AuNR stock solution (1 mL, 50 nM) and reacted for 24 h. NH_2_-PEG-SH (200 μL, 2.5 mM) was added to AuNR stock solution (1 mL, 50 nM) or deionized water (1 mL) and reacted under the same condition as positive and negative control groups, respectively. After 24 h reaction, mixtures were centrifuged (10,000 rpm, 15 min, RT). The unconjugated NH_2_-PEG-SH in the supernatants was collected and detected by Ninhydrin assay. Amine group content in NH_2_-PEG-SH in the supernatant of the positive group which was subtracted from the negative control group accounted for 100% conjugated PEG. The conjugated DTPA-PEG-SH in the experimental group was estimated as 50% of the total conjugated PEG. The DTPA-AuNR_PEG-NH2_ pellet was resuspended into 0.1% Tween® 20 and washed twice by centrifugation before radiolabelling.

### 2.5 Characterization

#### 2.5.1 UV–vis–NIR absorption

The optical properties of AuNRs and functionalized AuNRs were determined by UV–Vis spectroscopy using Lambda2 UV/ VIS Spectrometer (Perkin Elmer, USA) with a wavelength range of 400–1000 nm.

#### 2.5.2 Fourier transform infrared spectrum characterization

DTPA-AuNR_PEG-NH2_ was characterized using a Frontier FT-IR spectrometer (PerkinElmer, USA). Each sample was analyzed at RT in the spectral range of 4000–800 cm^−1^ with a total of 32 scans per run.

#### 2.5.3 Nanoparticle tracking analysis (NTA)

Hydrodynamic size and particle concentration of the functionalized AuNRs were determined by nanoparticle tracking analysis (NTA) using NanoSight LM10 (Malvern Instruments, UK). NTA represents the most reliable method to directly establish the nanoparticle concentration [38]. The particles were diluted with filtered deionized water to obtain 20–80 particles in the viewing frame. The modal size and particle count were measured in quadruplicate, with 30 s as the duration for each recording, and analyzed using the NanoSight NTA 3.2 software (Malvern Instruments, UK).

#### 2.5.4 Zeta potential

The Zeta potential of the particles was determined at RT by electrophoretic mobility measurement using Zetasizer Nano series (Malvern Instruments, UK).

#### 2.5.5 Transmission electron microscopy

The morphology of the particles was evaluated by transmission electron microscope (TEM). A drop of particle sample after purification was loaded onto a carbon-coated 300-mesh copper grid and allowed to stand for 3 min. The excess fluid was absorbed by filter paper. The grid was quickly washed with filtered deionized water and dried in air. The grids were then imaged at 200 kV with a JEM-2100 transmission electron microscope (JEOL, Japan). For the negative staining, the sample placed on the grid was treated with 3% uranyl acetate for 2–3 min. Excess fluid was removed by a filter paper, washed twice with filtered deionized water and dried in air then the grid was imaged. The obtained images were analyzed using ImageJ software (USA). More than 200 nanoparticles were counted for the length and width measurement.

### 2.6 Radiolabelling efficiency and radiochemical stability analysis

Radiolabelling efficiency of DTPA-PEG-SH was firstly assessed. In brief, 2 MBq (2.5–4 μL) of ^111^InCl_3_ stock was added to 0.2 M ammonium acetate buffer (pH 5.5) to achieve a final volume of 100 μL. The mixture was then added to an equal volume of DTPA-PEG-SH (∼1 mM in deionized water) to achieve a final concentration of 0.1 M ammonium acetate. The mixture was incubated for 30 min at RT by vortexing every 5 min in between. EDTA (5 μL, 0.1 M) was added to stop the reaction. Afterwards, 1 μL of the sample was spotted on an instant TLC (iTLC) paper strip. The strips were developed in 0.1 M ammonium acetate containing 0.25 mM EDTA (pH 5.5) as the mobile phase. The strips were then exposed to a multipurpose storage phosphor screen (Cyclone®, Packard, UK) in autoradiography cassettes for ∼5 min, analyzed on a Cyclone Storage Phosphor System and quantified using Optiquant software (Packard, Meriden, USA). The spots at the application point of the iTLC strips correspond to the ^111^In labelled DTPA-PEG-SH. Radiolabelling efficiency was calculated as % radioactivity remaining at the application point.

For DTPA-AuNR_PEG-NH2_ radiolabelling, the particles with the desired amount were resuspended in 0.1% Tween® 20 and mixed with the required amount of ^111^InCl_3_ stock, 0.5–1, 3–5, 5–10 MBq per mouse for gamma counting, autoradiography and SPECT/CT imaging, respectively, and reacted under the conditions described above. The radiolabelled DTPA-AuNR_PEG-NH2_ was purified by centrifugation at 10,000 rpm for 20 min at RT to remove free ^111^In prior to *in vivo* studies.

To detect DTPA or DTPA-PEG-SH contamination, DTPA-AuNR_PEG-NH2_ was spotted on the iTLC paper strip and run on 3.5% ammonia: methanol (1:1, *v*/v, pH 10.5) without EDTA as the mobile phase.

For radiochemical stability analysis, [^111^In]DTPA-AuNR_PEG-NH2_ was incubated in PBS or 50% serum for 24 h at RT or 37 °C and then spotted on iTLC paper strips. The strips were developed in 0.1 M ammonium acetate containing 0.25 mM EDTA (pH 5.5) as the mobile phase. The ^111^In remained conjugated to DTPA-AuNR_PEG-NH2_ (immobile spot at the application point) was considered as radio-chemically stable.

### 2.7 Animals

All animal experiments were performed in compliance with the UK Animals (Scientific Procedures) Act 1986 and UK Home Office Code of Practice for the Housing and Care of Animals Used in Scientific Procedures (Home Office 1989). *In vivo* experimentation was adhered to the project license approved by the King's College London animal welfare and ethical review body (AWERB) and UK Home Office (PBE6EB195). Female CD-1 mice (25–35 g, 6–8 weeks old) were obtained from Charles River (UK) for multi-modal tracking studies. Male and female C57BL/6 mice (18–25 g, 4–6 weeks old) obtained from Charles River (UK) were used for orthotopic glioblastoma mouse model establishment. Both sexes were included in the study in line of the new published recommendations by the Medical Research Council, UK, for conducting research on animals.

#### 2.7.1 Tumor model induction

Intracranial GL261 glioma model was established in C57BL/6 mice as described previously with modifications [39,40]. Female and male C57BL/6 mice, aging 4–6 weeks, were anesthetized using isoflurane inhalation. Prior to surgery, animals received a subcutaneous injection of 0.3 mg/kg of Vetergesic. The mice were then injected stereotactically with 200 K of GL261 murine glioma cells expressing Red-Fluc luciferase (BW134246, Perkin-Elmer) suspended in 2 μL PBS into the left hemisphere using a Hamilton syringe (Harvard Apparatus, UK) with a 28-gauge needle at a rate of 0.2 μL/min. The stereotactic coordinates relative to bregma were: 0.5 mm anterior, 1.5 mm lateral and 2.5 mm deep. Tumor growth was monitored by bioluminescence imaging twice a week (IVIS Lumina III, Perkin-Elmer, UK). Anesthetized mice were injected subcutaneously with 150 mg/kg luciferin (D-luciferin potassium salt, Perkin-Elmer, UK) and imaged 10 min after injection. Bioluminescence signals from the regions of interest were measured using Living Image software (Perkin-Elmer, UK) and recorded as total flux (photons/s). Animals were used for gamma counting biodistribution studies when the tumors reached the desired size ∼2 weeks after the implantation (total flux >1 × 10^7^ p/s).

### 2.8 *Ex vivo* optical imaging studies

Female CD-1 mice had food restriction but with free access to water for 24 h before administration for *ex vivo* optical imaging studies. Cy5-AuNR_PEG-NH2_ was suspended in CTS solution (0.5% CMC, 0.1% Tween® 20 and 0.9% NaCl, *w*/w) to ensure sufficient retention of the nanoparticles in the nasal passage. AuNR was administered at a final particle concentration of 300 nM. Mice were intranasally administered under inhalational anaesthesia with formulations by dosing 2 μL to the left and right nostrils alternatively at a minimum 20 s interval. A total of 20 μL were administered to each mouse. The administered AuNR was ∼6 pmol/mouse. At predetermined time points (10 min, 30 min, 1 h, 3 days and 7 days) post-administration, mice were culled by cervical dislocation without cardiac perfusion. Organs including brain, heart, lung, liver, spleen, kidneys, stomach and intestine were collected, weighed and imaged using an IVIS Lumina III system (PerkinElmer, UK). Mice without any treatment were used as control groups. Cy5 free dye dissolved in CTS solution was administered as a control at equivalent fluorescence intensity to the formulations.

Fluorescence images were obtained using Cy5 filter (Ex: 620 nm/Em: 670 nm) with the exposure time of 1 s for the major organs and 25 s for the separated brains. The obtained images were analyzed using the Living Image 4.7.2 software (PerkinElmer, UK) where the regions of interest (ROIs) were drawn for each organ to obtain the fluorescence signals.

### 2.9 ICP-MS measurement for brain uptake

After *ex vivo* optical imaging studies, brains were weighed using an analytical balance (Secura, Germany), followed by drying in an oven at 70 °C in 15 mL pre-cleaned trace metal grade HDPE centrifuge tubes. Afterwards, 1.5 mL HCl (37%, *w*/w) and 0.5 mL HNO_3_ (68%, w/w) as the composition of aqua regia were added to individual brain samples separately at 2–3 min intervals. Tubes were allowed to settle for 10 min at RT then were closed properly. Samples were digested in an oven at 70 °C overnight. After digestion, the tubes were vortexed slightly and centrifuged (4000 rpm, 40 min, RT) to precipitate the undissolved fat. To correct the instrument drift and matrix effects, 50 μL of 2 ppm Iridium (Ir), as the internal standard, was spiked into 250 μL of supernatants, deionized water was then added to a final of 5 mL for ICP-MS measurement (Perkin Elmer, UK). Gold calibration curve between 0.1 and 250 μg/L was established. All calibration solutions and blanks were doped with Ir as the internal standard. Quality control of ICP-MS measurements was ensured through repeated measurements of blanks and a calibrant.

### 2.10 Quantitative biodistribution of radiolabelled AuNRs using gamma counting

Organ biodistribution profiles of [^111^In]DTPA-AuNR_PEG-NH2_ were investigated in female CD-1 mice using gamma counting to obtain quantitative data. Mice were intranasally administered with 20 μL of 300 nM [^111^In]DTPA-AuNR_PEG-NH2_ in CTS solution (0.5–1 MBq). Blood sample (20 μL) was collected from the tail vein at 10 min, 30 min or 1 h post-administration. Mice were then sacrificed, organs and tissues such as skin, liver, spleen, kidneys, heart, lung, muscle, bone (femur), brain, stomach, intestine, nasal passage and carcass were collected, weighed, and placed in scintillation vials. The radioactivity of the samples was measured by a gamma counter (RUO WIZARD^2^ 2-detector 550 samples, PerkinElmer, UK) together with radioactive dose standards. To further investigate the brain distribution of the particles, brains were dissected into four coronal sections (1: the olfactory bulbs (OB), 2: front cerebrum (CB 1), 3: back cerebrum (CB 2), 4: brain stem (BS) and cerebellum (CE)).

To investigate AuNRs biodistribution in an orthotopic glioblastoma mouse model, at predetermined time points post-administration (10 min and 24 h), organs and tissues were collected as described above with the modification that brains were dissected to separate the tumor mass from the rest of brain tissue parenchyma. Organs were weighed using an analytical balance prior measurement of radioactivity using a gamma counter. Results were expressed as the percentage of injected dose per tissue (%ID/tissue) or percentage of injected dose per gram of tissue (%ID/g of tissue).

### 2.11 Autoradiography

To investigate the regional distribution of [^111^In]DTPA-AuNR_PEG-NH2_ in brains, CD-1 mice were intranasally administered with 20 μL of 300 nM [^111^In]DTPA-AuNR_PEG-NH2_ in CTS solution (3–5 MBq) for autoradiography. Brains were harvested at 10 min, 30 min or 1 h post-administration. Each brain was cut into 2 mm thick sagittal sections using a mouse brain slicer (Zivic-Miller, USA). Sections were placed between two glass microscope slides before exposing to a super-sensitive plate (Storage Phosphor Screen BAS-IP, Fujifilm, USA) in autoradiography cassettes for exposure. For brain harvested at 30 min or 1 h post-administration, these sandwich units were exposed for ∼40 h in the autoradiography cassettes. For brain harvested at 10 min post-administration, these sandwich units were allowed to decay for 2 days and then exposed for ∼16 h in the autoradiography cassettes before imaging using a laser scanner (Typhoon™ FLA 7000, GE Healthcare Life Sciences, UK). The obtained images were analyzed using ImageJ software (USA).

### 2.12 Whole-body SPECT-CT imaging

SPECT-CT imaging was performed using a VECTor^6^CT^XUHR^ preclinical small animal scanner (MILabs, The Netherlands). CD-1 mice were intranasally administered with [^111^In]DTPA-AuNR_PEG-NH2_ (20 μL of 300 nM [^111^In]DTPA-AuNR_PEG-NH2_ in CTS solution, 5–10 MBq), or the equivalent amount of radioactivity of [^111^In]EDTA or [^111^In]DTPA-PEG-SH. SPECT images were acquired at 0–30 min, 4 h and 24 h post-administration with a General Purpose Mouse collimator (GP-M, 0.6 mm pinhole size), followed by a CT scan. Throughout the imaging procedure, mice were kept under isoflurane anaesthesia in prone position on a heating pad to maintain the body temperature and the respiratory rate was monitored. All SPECT/CT images were reconstructed using MILabs reconstruction software v11.0 (MILabs, The Netherlands) and analyzed by VivoQuant 3.0 analysis software (inviCRO LLC, USA).

### 2.13 Statistical analysis

Quantitative results were presented as mean ± standard deviation (SD), where “n” denotes the number of repeats. Statistical differences were examined using one-way ANOVA, except for the gamma counting study for brain different sections in which two-way ANOVA was used, by GraphPad Prism 8 software (v 8.2.1). The *P* value <0.05 was considered statistically significant.

## 3 Results

### 3.1 Cy5-PEG-SH synthesis

**Scheme 1:**
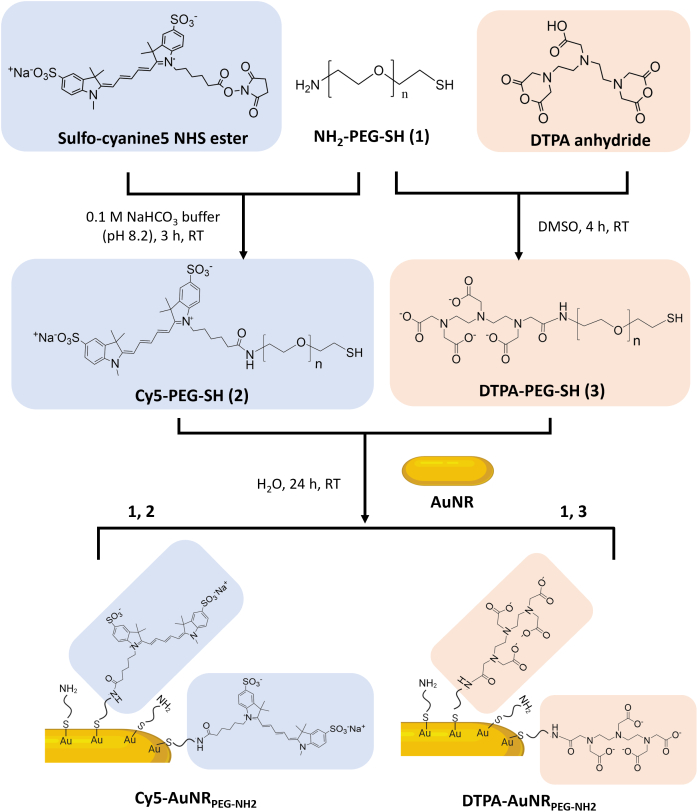
**Schematic illustrations representing the functionalization procedure of Cy5-AuNR**_**PEG-NH2**_**and DTPA-AuNR**_**PEG-NH2**_**.** Cy5-PEG-SH or DTPA-PEG-SH linkers were synthesized by the reaction between the NHS ester group of Cy5 or the anhydride of DTPA and the amine group of PEG. Cy5-PEG-SH or DTPA-PEG-SH was then conjugated with AuNRs through an Au—S bond to obtain Cy5-AuNR_PEG-NH2_ or DTPA-AuNR_PEG-NH2_.

Cy5-PEG-SH was synthesized by the reaction between the NHS ester group of Cy5 and the amine group of PEG to yield a stable amide bond **(**Scheme 1**)**. After synthesis, Cy5-PEG-SH was purified by the NAP™-5 column. Cy5-PEG-SH possessing a larger molecular weight was eluted first from fractions 3 to 7, confirmed by the strong fluorescence signals **(Fig. S1A** and **B)**. The same fractions were positive for PEG **(Fig. S1C)**, further confirming the successful synthesis of Cy5-PEG-SH. The unconjugated Cy5 were collected in fractions 13 to 24 **(Fig. S1A** and **B)**.

### 3.2 DTPA-PEG-SH synthesis

The free amine in PEG acts as a nucleophile which attacks the anhydride on the DTPA, resulting in an amide bond formation **(**Scheme 1**)**. To optimize the synthesis conditions, NH_2_-PEG-SH was reacted with DTPA anhydride at molar ratios of 1:2, 1:1, 2:1 and 4:1 using DMSO as solvent **(Table S1)**. It was shown that at the molar ratio of 1:1 (PEG: DTPA anhydride), ∼75.4% of amines in PEG were substituted after 4 h reaction. By increasing the concentration of DTPA anhydride to the molar ratio of 1:2 (PEG: DTPA anhydride) or extending the reaction time to 24 h, there was no significant improvement in the PEG substitution. It is worth noticing that when NH_2_-PEG-SH was reacted with DTPA anhydride at the molar ratio of 4:1, ∼20.8% of amines in PEG were substituted after 4 h and the PEG substitution was increased to ∼43.8% after 24 h, indicating disubstitution with DTPA anhydride may have occurred. Therefore, the molar ratio between NH_2_-PEG-SH and DTPA anhydride of 1:1 and the reaction time of 4 h were applied for large-scale synthesis. In the final products, the proportion of DTPA fragments to PEG fragments quantified by the modified Gd^3+^-Xylenol Orange assay and iodine solution-based assay, respectively, was in the range of 0.8–1, confirming the successful synthesis of DTPA-PEG-SH.

### 3.3 Characterization of Cy5-AuNR_PEG-NH2_ and DTPA-AuNR_PEG-NH2_

**Fig. 1:**
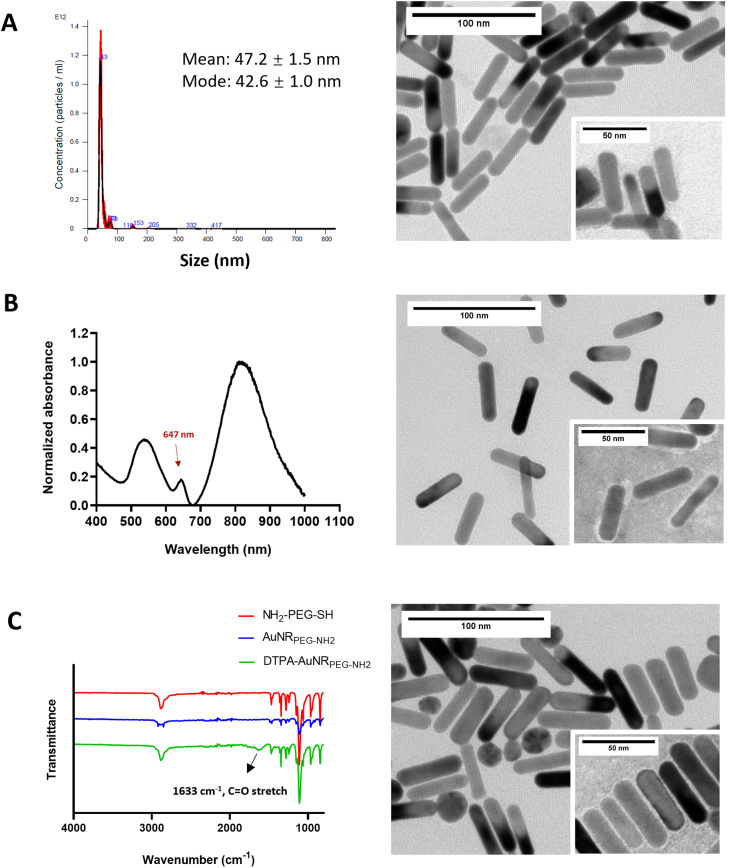
**Characterization of AuNR conjugates**_**.**_**(A)** Representative hydrodynamic size distribution and TEM images of AuNR_PEG-NH2._**(B)** Representative UV–vis–NIR spectrum and TEM images of Cy5-AuNR_PEG-NH2._ The conjugated Cy5-AuNR_PEG-NH2_ demonstrated a typical peak which is consistent with the excitation wavelength of Cy5. **(C)** FT-IR spectra and TEM images of DTPA-AuNR_PEG-NH2._ The typical PEG bonds and the amide bond at 1633 cm^−1^ in DTPA-AuNR_PEG-NH2_ confirmed the successful DTPA-PEG-SH conjugation to AuNRs. TEM insets represent uranyl acetate-stained TEM images.

Cy5-PEG-SH was conjugated to AuNRs through an Au—S bond. The UV–vis–NIR spectrum of Cy5-AuNR_PEG-NH2_ after synthesis demonstrated a typical absorbance at 647 nm **(**Fig. 1 and **Fig. S2B)**, which matched the excitation wavelength of Cy5, indicating the successful synthesis of Cy5-AuNR_PEG-NH2_. After synthesis, 1305 ± 406 Cy5-PEG-SH molecules were conjugated with single AuNRs. Cy5-AuNR_PEG-NH2_ showed a narrow particle size distribution with the hydrodynamic size of 85.6 ± 14.9 nm determined by NTA. The zeta potential of Cy5-AuNR_PEG-NH2_ was 24.0 ± 0.3 mV.

The FT-IR spectrum of DTPA-AuNR_PEG-NH2_ demonstrated the typical bonds of PEG and an amide bond at 1633 cm^−1^, confirming the successful DTPA-PEG-SH conjugation to AuNRs **(**Fig. 1**, Fig. S2C)**. In the positive control group, 3267 ± 938 PEG molecules were conjugated to a single AuNR determined by Ninhydrin assay. Therefore, it was estimated that each AuNRs is conjugated to ∼1600 DTPA-PEG-SH molecules. Compared with AuNR_PEG-NH2,_ DTPA-AuNR_PEG-NH2_ demonstrated an increased hydrodynamic size of 64.0 ± 3.4 nm and a decreased Zeta potential of 11.4 ± 0.5 mV due to the reduced amine content on the particle surface. Both Cy5-AuNR_PEG-NH2_ and DTPA-AuNR_PEG-NH2_ demonstrated good colloidal stability. The physicochemical characteristics of Cy5-AuNR_PEG-NH2_ and DTPA-AuNR_PEG-NH2_ are summarized in Table 1.

**Table 1:** Physicochemical characterization of Cy5-AuNR_PEG-NH2_ and DTPA-AuNR_PEG-NH2._

Compound	PEG ^[1]^	Cy5 ^[2]^	DTPA ^[3]^	Size ^[4]^	Zeta potential ^[5]^
	graft ratio (mole: mole AuNRs)			(nm)	(mV)
AuNR_PEG-NH2_	3267 ± 938	–	–	47.2 ± 1.5	42.6 ± 0.9
Cy5-AuNR_PEG-NH2_		1305 ± 406	–	85.6 ± 14.9	24.0 ± 0.3
DTPA-AuNR_PEG-NH2_		–	∼1600	64.0 ± 3.4	11.4 ± 0.5

[1] Determined by indirect Ninhydrin assay; [2] Measured by plate reader; [3] Estimated as 50% of the total conjugated PEG; [4] Measured by nanoparticle tracking analysis (NTA); [5] Measured by Zetasizer Nano series.

Representative TEM images of the particles are shown in Fig. 1 and **Fig. S3**. The images show no apparent structural differences after Cy5 or DTPA functionalization. All the particles demonstrated monodispersed rod morphology with the length of 45.1 ± 4.4 nm and width of 11.2 ± 1.3 nm (>200 particles counted).

### 3.4 *Ex vivo* biodistribution of AuNRs by optical imaging

**Fig. 2:**
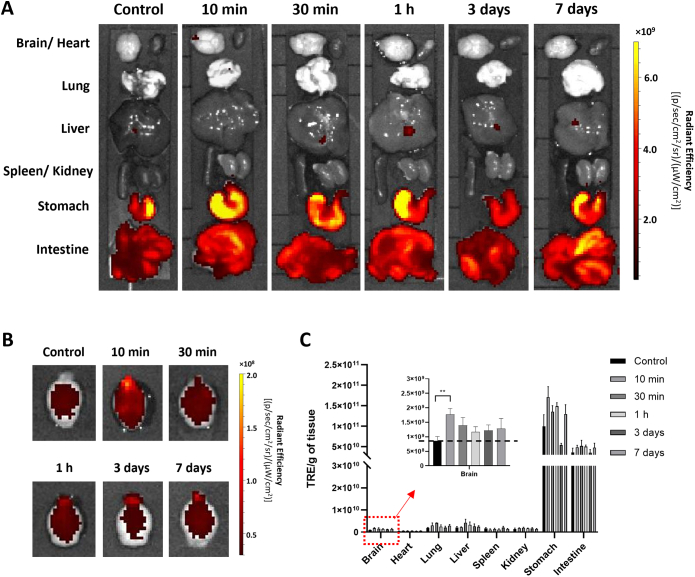
***Ex vivo* imaging of Cy5-AuNR**_**PEG-NH2**_**in CD-1 mice after intranasal administration.** Cy5-AuNR_PEG-NH2_ (300 nM, 20 μL) was intranasally administered into CD-1 mice. Representative *ex vivo* images of excised **(A)** organs and **(B)** brains harvested at 10 min, 30 min, 1 h, 3 days and 7 days post-administration. Mice without any treatment were used as the control group. **(C)***Ex vivo* quantification of fluorescence signals of Cy5-AuNR_PEG-NH2_ per gram of tissue at different time points. Values were expressed as mean ± SD, *n* = 3. **P < 0.01. All images were obtained by IVIS Lumina® III and data were analyzed by Living Image software.

*Ex vivo* organ distribution profiles were first assessed by optical imaging using Cy5-labelled AuNRs. Mice were intranasally administered with 20 μL of 300 nM nanoparticles. At this dose, mice behaved normally without change in the body weight up to 7 days post-administration **(Fig. S4)**. Mice had food restriction but free access to water for 24 h before experiments to reduce food interference in optical imaging. High autofluorescence signals particularly in the stomach and intestine were seen in control and treated mice **(**Fig. 2A**)** making it hard to distinguish if the signals in stomach/intestine are attributed to AuNRs or an artefact of tissue background. Similar results have been reported previously where extracellular vesicles were bioengineered with mCherry (ex/em = 587 nm/610 nm) to track their *ex vivo* organ distribution profiles [41]. Interestingly, in *ex vivo* imaged brains **(**Fig. 2B and C**)**, higher fluorescence signals were observed in treated mice compared with the control mice, especially at 10 min post-administration (**P < 0.01). The highest fluorescence signal was seen in the frontal brain region. To confirm that the detected fluorescence signals represented Cy5-AuNR_PEG-NH2_, Cy5 molecules alone with identical fluorescence intensity were administered into the CD-1 mice which have shown similar signals intensity in the brain to that of control mice (*P* > 0.05) suggesting that increased fluorescence signals was indeed attributed to AuNRs translocation to the brain **(Fig. S5)**.

### 3.5 ICP-MS measurement for gold brain uptake

**Fig. 3:**
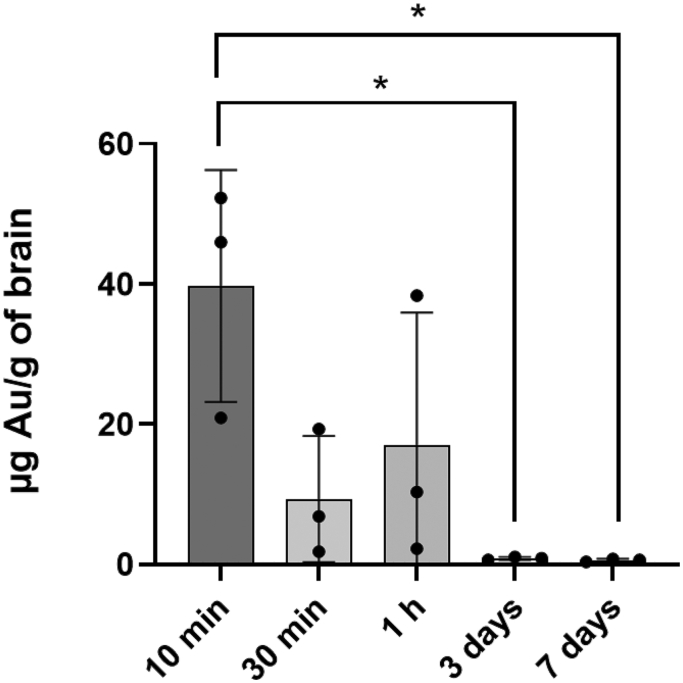
**ICP-MS analysis of brain Au contents.** Cy5-AuNR_PEG-NH2_ (300 nM, 20 μL) was intranasally administered into CD-1 mice and brains were harvested at 10 min, 30 min, 1 h, 3 days and 7 days post-administration. The total amount of Au in the brain tissue after digested by aqua regia was measured by ICP-MS. Values were expressed as mean ± SD, *n* = 3. *P < 0.05.

Although the Au—S bond can be considered as a covalent bond with the bond energy of 40–50 kcal/mol [42,43], it was reported the monothiol ligand may experience a high dynamic off-rate that destabilizes the particles in biologically relevant reducing environments [44]. To further confirm the fluorescence signals detected in the brain came from the intact Cy5-AuNR_PEG-NH2_ rather than the detached Cy5-PEG, the brain samples after *ex vivo* optical imaging were proceeded for ICP-MS to quantify the gold content. Brains harvested at 10 min post-administration showed the highest Au uptake, achieving 39.71 ± 16.57 μg Au/g of brain **(**Fig. 3**)**. At later time points 30 min and 1 h post-administration, Au uptake in brains was reduced. After 3 days and 7 days administration, the Au contents in brains were significantly reduced to 0.86 ± 0.22 and 0.59 ± 0.21 μg Au/g of brain, respectively (*P < 0.05 *vs* 10 min post-administration). Au contents in the control brains were ∼ 0.09 μg Au/g of brain. These findings are consistent with the trend observed by the optical imaging that AuNRs distributed rapidly to the brain except that Au could be detected in brains at 30 min and 1 h presumably due to the high sensitivity of the technique and the fact that ICP-MS measures Au directly.

### 3.6 Radiolabelling efficiency and radiochemical stability of DTPA-AuNR_PEG-NH2_

**Fig. 4:**
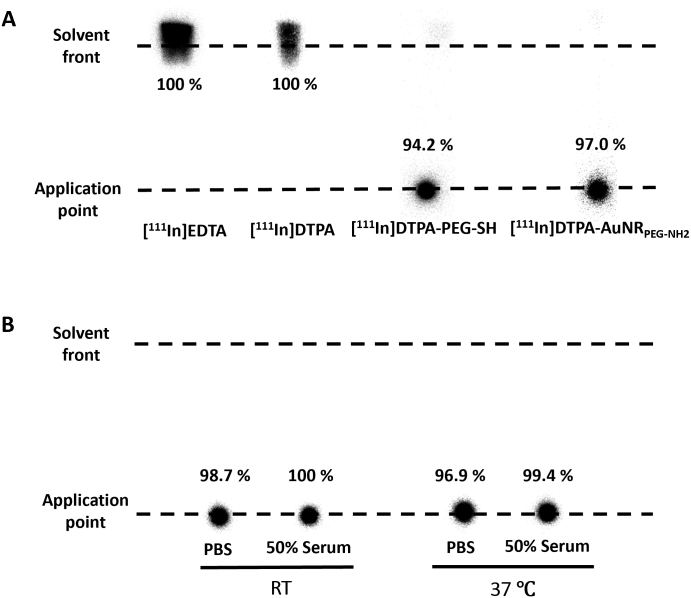
**Radiolabelling efficiency and radiochemical stability of DTPA-AuNR**_**PEG-NH2.**_**(A)** Radiolabelling efficiency of DTPA-PEG-SH and DTPA-AuNR_PEG-NH2_. **(B)** Radiochemical stability of DTPA-AuNR_PEG-NH2._ [^111^In]DTPA-AuNR_PEG-NH2_ were incubated in PBS or 50% serum for 24 h at room temperature (RT) or 37 °C, and then spotted on a iTLC paper. The paper was then run on 0.1 M ammonium acetate with 0.25 mM EDTA (pH 5.5) as the mobile phase and imaged using a phosphorimager. Radiolabelling efficiency and radiochemical stability were calculated as % radioactivity remaining at the application point.

Quantitative biodistribution assessments were also carried out by radiolabelling of AuNRs with the gamma emitting radioisotope ^111^In and studying the biodistribution of [^111^In]DTPA-AuNR_PEG-NH2_ construct. Radiolabelling efficiency of DTPA-PEG-SH was first assessed using iTLC by eluting with 0.1 M ammonium acetate containing 0.25 mM EDTA (pH 5.5). Free DTPA chelated with ^111^In migrated to the solvent front and the radiolabelled DTPA-PEG-SH remained at the application point **(**Fig. 4A and **Fig. S6)**. DTPA-PEG-SH conjugates after synthesis were collected through PD-10 columns, followed by different times of elution. The radiolabelling efficiency of DTPA-PEG-SH conjugates increased from 18.0% to 71.2% and 94.2% after the first, second and third elution, respectively **(Fig. S6)**. The DTPA-PEG-SH after the third elution were used for AuNRs functionalization. The resulting DTPA-AuNR_PEG-NH2_ demonstrated radiolabelling efficiency of 97% as shown in Fig. 4A.

To further test the contamination of DTPA or DTPA-PEG-SH in DTPA-AuNR_PEG-NH2,_ the particles after radiolabelling were spotted on iTLC and run on 3.5% ammonia: methanol (1:1, *v*/v, pH 10.5) as the mobile phase. In this condition, DTPA or DTPA-PEG-SH after complexing with ^111^In migrated to the solvent front **(Fig. S7)**. [^111^In]DTPA-AuNR_PEG-NH2_ demonstrated a migration from the application point without presenting significant radioactivity in the solvent front. The above results confirmed the high radiolabelling efficiency of [^111^In]DTPA-AuNR_PEG-NH2_ with a negligible amount of free DTPA or DTPA-PEG-SH. Also, DTPA-AuNR_PEG-NH2_ demonstrated good radiochemical stability (> 95%) both in PBS and 50% serum after 24 h either at RT or 37 °C **(**Fig. 4B**)**.

### 3.7 Whole body distribution of AuNRs by SPECT/CT imaging

**Fig. 5:**
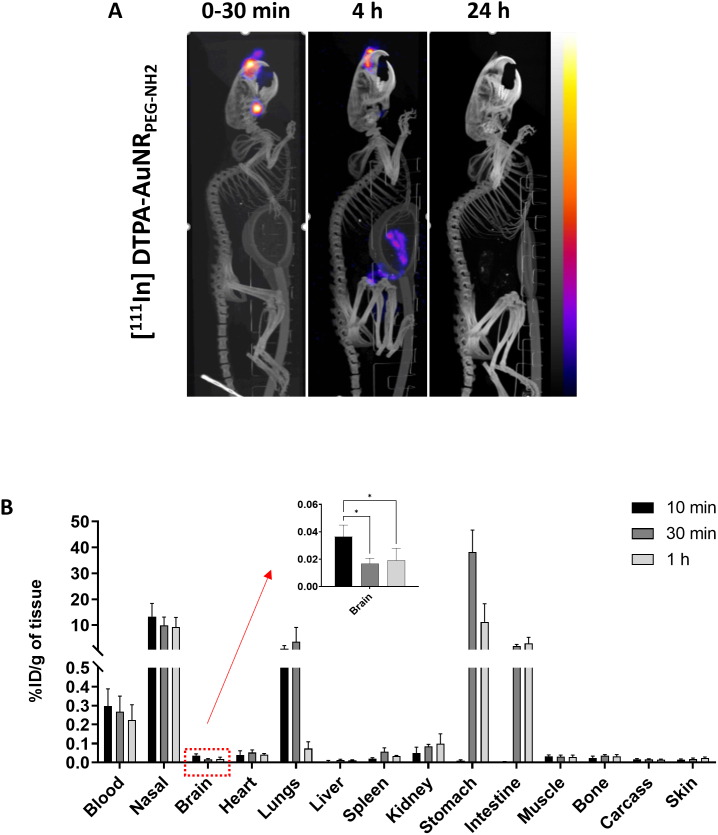
**Whole body biodistribution profiles of [**^**111**^**In]DTPA-AuNR**_**PEG-NH2**_**in CD-1 mice after intranasal administration. (A)** Whole body SPECT/CT imaging of [^111^In]DTPA-AuNR_PEG-NH2_ in CD-1 mice. CD-1 mice were intranasally administered with 5–10 MBq of [^111^In]-labelled compounds. Imaging was done at 0–30 min, 4 h and 24 h post-administration. **(B)** Organ biodistribution of [^111^In]DTPA-AuNR_PEG-NH2_. CD-1 mice were intranasally administered with 0.5–1 MBq of [^111^In]-labelled compounds and were culled at 10 min, 30 min or 1 h post-administration. Inset shows the zoomed-in %ID per gram of brain uptake. Values were expressed as mean ± SD, *n* = 3. *P < 0.05.

SPECT/CT uses ionizing radiations for imaging which does not suffer from tissue penetration limitations encountered in optical imaging. SPECT/CT images were therefore more reliable for interpretation of AuNRs uptake in internal organs. Whole body distribution of AuNRs was studied by SPECT/CT imaging up to 24 h post administration. [^111^In]DTPA-AuNR_PEG-NH2_ and [^111^In]DTPA-PEG-SH demonstrated longer retention time in nasal passage than free [^111^In]EDTA **(Fig. S8)**. After 4 h intranasal administration, majority of [^111^In]DTPA-AuNR_PEG-NH2_ remained in nasal passage with part of the dose transiting to the gastrointestinal (GI) tract. In comparison, the majority of unconjugated [^111^In]EDTA was detected in lower GI tract. Negligible signals were observed in the body at the 24 h timepoint for all groups **(**Fig. 5A and **Fig. S8)**.

### 3.8 Quantitative organ distribution of AuNRs by gamma counting

To gain quantitative insight on biodistribution of [^111^In]DTPA-AuNR_PEG-NH2_, gamma counting was performed on all organs. For all groups, ∼ 30% of injected dose (ID) remained in the nasal passage **(Fig. S9)** after intranasal administration. Radioactivity in the stomach increased from 0.009 ± 0.005%/g of tissue at 10 min to 38.2 ± 8.5%/g of tissue at 30 min and then decreased to 11.2 ± 7.0%/g of tissue at 1 h post-administration **(**Fig. 5B**)**. Similarly, the radioactivity in the intestine increased from 0.003 ± 0.002 %ID/g of tissue at 10 min to 2.0 ± 0.5 and 3.0 ± 2.3% ID/g of tissue at 30 min and 1 h post-administration, respectively. In contrast, [^111^In]DTPA-AuNR_PEG-NH2_ showed noticeable accumulation in the lung with 0.87 ± 1.21 and 3.6 ± 5.5% ID/g of tissue at 10 min and 30 min, respectively, which dropped to 0.07 ± 0.04% ID/g of tissue at 1 h post-administration. This suggests that some particles went beyond nasopharynx and got into lung after administration through a process of mucociliary clearance. Most of the particles were swallowed by the mice and went through the GI system in the later time points (> 10 min). This property allows the particles to be excreted from the animal through faeces, leading to minimized systemic exposure. Other major organ biodistribution profiles of [^111^In]DTPA-AuNR_PEG-NH2_ at 1 h post-administration showed the uptake in kidneys > heart> spleen > liver with 0.10 ± 0.05, 0.04 ± 0.007, 0.03 ± 0.004 and 0.01 ± 0.002 %ID/g of tissue, respectively. In addition, ∼ 0.6% of ID was found in the blood throughout **(Fig. S9)**, suggesting a small amount of the particles can be rapidly absorbed into the systemic circulation after intranasal administration. Looking at brain accumulation, 10 min post-administration demonstrated the highest brain uptake with 0.036 ± 0.008% ID/g of tissue (*P < 0.05, 10 min *vs* 30 min and 1 h post-administration). Brain accumulation decreased to 0.019 ± 0.009% ID/g of tissue at 1 h post-administration.

### 3.9 Brain regional distribution of AuNRs by autoradiography

**Fig. 6:**
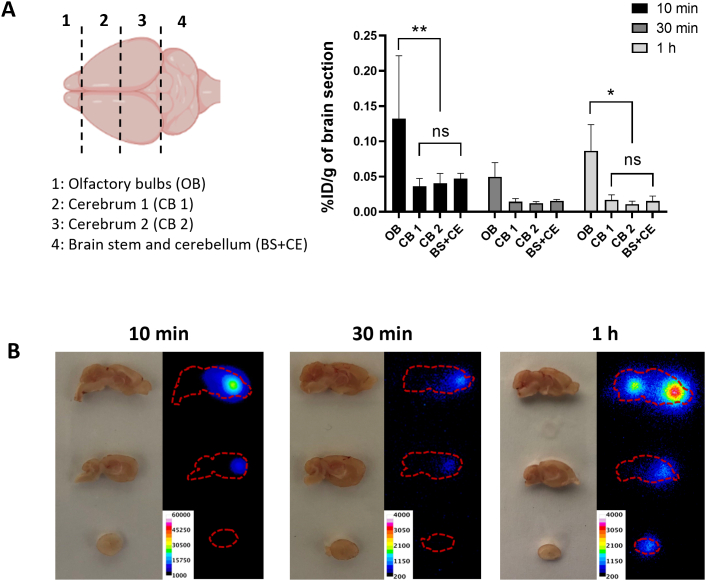
**Brain region-specific distribution of [**^**111**^**In]DTPA-AuNR**_**PEG-NH2**_**in CD-1 mice after intranasal administration. (A)** [^111^In]DTPA-AuNR_PEG-NH2_ brain uptake. Brains were dissected into four coronal sections. Values were expressed as mean ± SD, n = 3. *P < 0.05, **P < 0.01. **(B)** Autoradiographs of mouse brain. Mice were intranasally administered with 3–5 MBq of [^111^In]DTPA-AuNR_PEG-NH2_. Brains were harvested at 10 min, 30 min or 1 h post-administration and sectioned in sagittal orientation in 2 mm thick sections.

To further investigate the brain distribution of the particles, brains were cut into four coronal segments **(**Fig. 6A**)**. The results showed that the highest brain uptake was detected in the olfactory bulbs at all time points tested at 3–8 folds values obtained in other coronal brain sections.

Autoradiography was then applied to provide direct evidence of the region-specific brain accumulation of [^111^In]DTPA-AuNR_PEG-NH2_. The exposure time has been adjusted depending on the amount of radioactivity for each group for better illustration. Brain harvested at 10 min post-administration demonstrated the highest signals among all time points **(**Fig. 6B**)**. These findings matched the previous results in optical imaging, ICP-MS and gamma counting. Interestingly, with time increasing to 1 h post-administration, a clear signal was noticed in the caudal brain, indicating the deeper transport of the [^111^In]DTPA-AuNR_PEG-NH2_ into brain regions with time.

### 3.10 AuNRs brain uptake in glioblastoma bearing mice

**Fig. 7:**
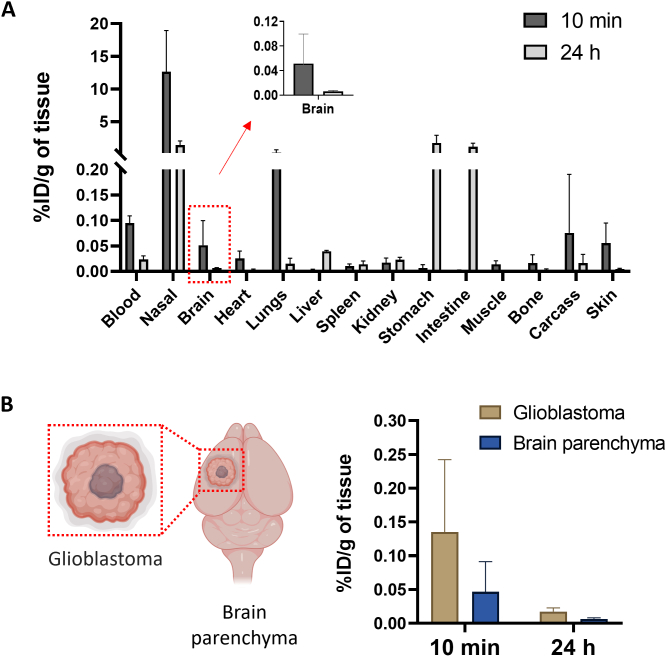
**Organ biodistribution profiles of AuNR in C57BL/6 glioblastoma bearing mice after intranasal administration. (A)** Organ biodistribution of [^111^In]DTPA-AuNR_PEG-NH2_. Animals were culled at 10 min or 24 h post-administration. Inset: %ID per gram of entire brain. **(B)** Uptake in glioblastoma and brain parenchyma of dissected brains at 10 min or 24 h post-administration. Values were expressed as mean ± SD, *n* = 3.

After confirming the ability of AuNRs to reach the brain after intranasal administration, we tested the hypothesis if AuNRs can reach brain tumors *via* the nasal route. C57BL/6 mice were intracranially implanted with GL261 tumor cells. The tumor growth curves after GL261 cells injection are shown in **Fig. S10**. When the tumors reached the desired size, mice were intranasally administered with [^111^In]DTPA-AuNR_PEG-NH2_. At 10 min and 24 h post-administration, GL261 tumors were excised, AuNRs uptake was assessed by gamma counting and compared to the uptake in brain parenchyma of the same brains. The whole organ biodistribution profile at 10 min was similar to that obtained in healthy CD-1 mice **(**Fig. 7A**)**. At 24 h, uptake was dramatically reduced in nasal passage, blood, heart and lung. However, the AuNRs levels have increased in stomach, intestine and liver. The measured radioactivity in healthy C57BL/6 mouse brain at 10 min timepoint was lower (∼0.01% ID/g of tissue) than those values measured in CD-1 mice (∼0.036% ID/g of tissue) in agreement with other studies we performed using other types of carriers (data not shown). No significant difference in radioactivity was found between tumor tissues and brain parenchyma (*P* > 0.05) **(**Fig. 7B**)**. At 24 h, AuNRs were almost entirely cleared from both tumors and the brains. Altogether, the results confirmed the presence of AuNRs in brain tumors broadening the applications of intranasal AuNRs to include brain cancer indications in addition to neurodegenerative diseases.

## 4 Discussion

When designing effective nanomedicines, the shape of the particles has been considered as one of the most critical factors which can influence their cellular internalization and *in vivo* biodistribution [20,45]. Nanoparticles with a high AR tend to be taken up by cells at a faster rate and to a greater extent than particles with a low AR [46]. It was reported that for two similar sized particles, the particles with AR value of 3 showed 4-fold more efficient cell internalization compared to the particles with AR value of 1 [47]. Another study has shown more efficient extravasation and deeper penetration of AuNRs than nanospheres of the same effective hydrodynamic size in tumors [48]. This informed the basis of this study where we aimed to synthesize high rod purity AuNRs with an average AR value of 4 as a candidate for drug delivery carrier. The aim of this study is to come up with a conclusive overview on spatial and temporal brain distribution of AuNRs after intranasal administration as both the delivery system and the route of administration have recently attracted great attention in the field of non-invasive delivery to the brain in attempt to treat a range of CNS diseases including brain cancer [49,50].

Optical imaging uses non-ionizing radiation ranging from ultraviolet to infrared light to capture detailed images of tissues, cells and even molecules [51,52]. This imaging modality is highly desirable in the clinical and pre-clinical studies due to its safety, rapid screening and cost-effectiveness [51,53]. AuNPs are known as fluorescence quenchers. In principle, the quenching property of AuNPs is mainly dependent on three factors: morphology, their optical properties and the distance between the fluorescent dyes and the AuNPs. Efficient quenching occurs for gold nanospheres possessing a small diameter (<50 nm), a short distance to the fluorescent dyes (< 2 nm) and a plasmon resonance overlapping with the dye emission [54]. In this study, to diminish the quenching property of AuNRs, a PEG linker was introduced and a fluorescent dye (Cy5) with ∼660 nm emission wavelength best evading the plasmon resonances of the AuNRs was selected to enable AuNR tracking by optical imaging. A similar strategy has been successfully applied in another study where Alexa Fluor 647 with an emission wavelength of ∼670 nm was used to fluorescently label Angiopep-2-AuNR conjugates to assess their ability to improve blood-brain barrier crossing *in vitro*. The mechanism of cell internalization of the particles was investigated on bEnd.3 cells and studied by flow cytometry [27]. Optical imaging in the selected absorption window, visible spectrum instead of near infrared region, has its own limitations such as interference from tissue autofluorescence and tissue absorption, making deep tissue imaging more challenging and only semi-quantitative [32,33]. The variability observed in optical imaging of brains could be a combination of the semi-qualitative nature of the technique and the type of administration route. High variations in *in vivo* biodistribution studies are commonly observed in intranasal administration studies, in comparison for example to intravenous administration which can almost ensure 100% deposition of the substance into the bloodstream. Despite the individual variations, the observation from each modality is conclusive that AuNRs demonstrated rapid brain uptake which occurs within minutes and the signal decreased with time function. For the organs such as brains and lungs which are less affected by the background autofluorescence, optical imaging remains a facile approach to semi-quantitatively track the AuNR particles *ex vivo*. In this study, optical imaging was employed as the first-choice screening technique, and upon comparison with other imaging modalities, it was suggested that it should be employed in combination with at least one other imaging/quantification technique.

SPECT/CT images are obtained by reconstructing a series of imaging frames acquired over a period of time so that the 3D reconstructed image is an average of uptake over ∼30 min. SPECT/CT imaging done at 0–30 min, 4 h and 24 h was applied to visualize the long-term distribution and body clearance of AuNRs following intranasal administration. As shown in **Fig. S9**, within 1 h post-administration, most of the radioactivity remains in the nasal passage. Compared with high radioactivity in nasal passage (∼30% ID), the radioactivity in brains is too low (0.01–0.02% ID) to be clearly detected by SPECT imaging. The signal to noise ratio is what makes some organs appear brighter than others. A similar phenomenon was also observed by other researchers [55,56], in which the brain signals of ^123^I labelled-peptides and ^99m^Tc-labelled exosome were not shown in SPECT/CT images following intranasal administration, while brain uptakes were confirmed by gamma counting and autoradiography studies. The gamma counting and autoradiography were also used in this study to spatiotemporally track AuNRs brain distribution with higher detection sensitivity.

The autoradiographs showing the distribution of AuNRs in different brain regions strongly suggest the involvement of multiple transport pathways after intranasal administration. The olfactory pathway (olfactory nerve and olfactory epithelium) and trigeminal nerve pathway are primarily responsible for the nose to brain delivery [6,49,57,58]. The neural projections of the olfactory bulb extend into multiple rostral brain tissues such as the olfactory tract, anterior olfactory nucleus and piriform cortex so particle distribution in brain areas neighbouring the olfactory bulb observed over 10 min to 1 h period could be attributed to the olfactory pathway [57]. The distribution in the more distal regions at 1 h post-administration is possibly facilitated *via* the trigeminal nerve-mediated transport as the trigeminal nerve branches innervate the respiratory and olfactory epithelium, and also enter the brain stem in the pons [57,58].

Most of the studies reporting on gold nanostructures' biodistribution focus on intravenous route as mainstream administration method. Talamini et al. compared organ distribution of spherical-shaped (50 nm), rod-shaped (length: 60 nm, width: 30 nm) and star-shaped (55 nm as average of the longest tip-to-tip distance) gold nanostructures. No evidence of gold accumulation was found in brains after 1, 24, or 120 h by ICP-MS following intravenous injection [38]. Jong et al. investigated tissue distribution of AuNPs with spherical morphology in the size range of 10 nm to 250 nm after intravenous injection in rats [30]. They demonstrated that AuNPs with the size of 10 nm were detectable in all the evaluated tissues (blood, liver, spleen, kidney, heart, lung, testis and thymus) including the brain (∼ 0.3% of ID) at 24 h post-injection determined by ICP-MS. Larger particles (50 nm, 100 nm and 250 nm) could be detected in liver, spleen and blood but were excluded from the brain. Sonavane et al. also confirmed the ability of 15 nm and 50 nm AuNPs to reach the brain at 24 h timepoint after intravenous injection while 200 nm AuNPs showed negligible accumulation in tissues including brain, blood, stomach and pancreas [59]. These studies used single injection without active targeting. A plausible explanation could be that AuNPs with small particle <20 nm diameters can pass through the gap separating the astrocytic end-feet from the capillary endothelium [59,60], components of the brain microvascular units in addition to pericytes, astrocytes, tight junctions, neurons, and basal membrane [3].

Few studies have been carried out to investigate gold nanostructures organ biodistribution after intranasal administration. Ye et al. compared intravenous and intranasal administrations of gold nanocluster (∼ 5.6 nm) at 1 h timepoint [5]. The intravenous group showed >10-fold higher blood, lung, liver, spleen, kidney, and heart uptake compared to the intranasal group. No significant difference between the groups was found in the brain quantified by gamma counting. Brain uptake for the intranasal group was improved when focused ultrasound combined with microbubble-mediated technique was applied. To increase affinity to β-amyloid, a therapeutic target in Alzheimer's disease, Gallardo-Toledo et al. prepared D1-peptide functionalized gold nanospheres (∼47 nm) and investigated their biodistribution profiles [61]. They first demonstrated the brain accumulation achieved highest of 106 ± 19 ng Au/g tissue at 0.75 h and then decreased dramatically at 2, 4, 8 and 24 h timepoint after intranasal administration determined by Neutron Activation Analysis (NAA). However, only 1.9 ± 0.9 ng Au/g tissue achieved intravenously at 0.75 h timepoint, indicating a fast and significant delivery of AuNPs to CNS could be achieved using intranasal administration. In addition, they evaluated the brain distribution at 0.75 h timepoint using GoldEnhance™ kit for light microscopy after dissecting the brain in coronal sections and found a high percentage of the nanoparticles was in the olfactory bulb, periaqueductal gray, perirhinal and entorhinal cortex, and hippocampus region after intranasal administration. In case of intravenous administration, a greater percentage was observed in the basal forebrain, thalamus, and cerebellum. In our study, we achieved ∼40 μg Au/g of brain after 10 min post-administration which decreased to ∼9 and ∼ 17 μg Au/g of brain after 30 min and 1 h, respectively. It is worth noting that both size and shape are different in our study from the reported study.

Other groups have attempted to deliver gold nanoparticles and their hybrid materials to brain tumors by intranasal delivery but to our knowledge no work has been carried out on AuNRs using this route of administration. Wang et al. developed an intranasal anti-EphA3 functionalized AuNPs for temozolomide delivery to glioblastoma [12] which was shown to prolong the median survival time and increase apoptosis compared to free drug. ICP-MS and histological examinations showed reduced gold content in brain after 24 h and no visible damage was caused in major organs, respectively. Sukumar et al. have shown that intranasal therapeutic microRNAs loaded gold–iron oxide nanoparticles combined with systemic temozolomide improved the survival rate of glioblastoma bearing mice [62]. The presence of the Cy5-labbelled miRNA in brain cancer cells was also observed providing evidence that the therapeutic agent reached the tumor mass after intranasal administration. In our study, to investigate if AuNRs can exhibit an enhanced permeation and retention effect in the glioblastoma tumors which normally occurs after longer periods (∼4-24 h), we examined brain uptake after 24 h in addition to the 10 min timepoint. AuNRs achieved glioblastoma and brain parenchyma uptake of ∼0.14% ID/g of tissue and ∼ 0.05% ID/g of tissue, respectively, at 10 min. AuNRs could have their way to the tumors through the nose-to-brain route or from the systemic circulation *via* the respiratory mucosa since the blood brain barrier is compromised in glioblastoma tumors [63].

Cheng et al. conjugated doxorubicin (Dox) to transcription (TAT) peptide functionalized AuNPs [64]. This nanoconjugates achieved a brain concentration of ∼4.5 μg Au/g of tissue and demonstrated significant survival benefit compared to the free Dox after a single intravenous injection. The brain concentration of AuNRs achieved in this study is therefore expected to be therapeutically meaningful. Generally, AuNPs was considered bioinert and demonstrated negligible *in vivo* toxicity [65,66]. However, studies found intracranially injected AuNPs, especially with small particle size, could increase nestin expression which is related to CNS injury [67]. The biocompatibility of AuNRs following nose to brain delivery needs to be fully investigated in advancing them for therapeutic application.

## 5 Conclusions

AuNRs were successfully synthesized and functionalized to enable *ex vivo* and *in vivo* analyses in mice tissues. This is the first study to comprehensively analyze the brain region-specific accumulation of AuNRs following intranasal administration providing qualitative and quantitative insights using a battery of complementary techniques namely optical imaging, ICP-MS, gamma counting and autoradiography. The results demonstrated rapid brain uptake of AuNRs occurs within minutes of nasal instillation followed by gradual distribution to other brain regions over 1 h in healthy mice. Intranasal administration to an orthotopic glioblastoma mouse model confirmed that uptake of AuNRs extends to brain tumors in the same brains. Autoradiography images and uptake pattern suggested the involvement of olfactory and trigeminal pathways in brain uptake. The current study does not only provide qualitative and quantitative information about AuNRs uptake in the brain after intranasal administration for the first time but also confirmed the potential of AuNRs as intranasal delivery carriers to treat brain diseases including brain cancer.

## CRediT authorship contribution statement

**Shunping Han:** Methodology, Investigation, Writing – original draft, Funding acquisition. **Julie Tzu-Wen Wang:** Methodology, Investigation, Writing – review & editing. **Emine Yavuz:** Methodology, Investigation, Writing – review & editing. **Alaa Zam:** Methodology, Investigation. **Nadia Rouatbi:** Methodology, Investigation. **Rifka Nurul Utami:** Methodology. **Revadee Liam-Or:** Methodology. **Alexander Griffiths:** Methodology, Investigation. **Wayne Dickson:** Writing – review & editing. **Jane Sosabowski:** Resources, Writing – review & editing. **Khuloud T. Al-Jamal:** Conceptualization, Supervision, Funding acquisition, Writing – review & editing.

## Declaration of Competing Interest

The authors have declared that no competing interest exists.

## Data Availability

Data will be made available on request.

## Appendix A Supplementary data

Supplementary material Image 1
