# Supplementary material for: Spatiotemporal tracking of gold nanorods after intranasal administration for brain targeting
Source: J Control Release. 2023 May;357:606–19. doi: 10.1016/j.jconrel.2023.04.022 (PMC10390340; doi:10.1016/j.jconrel.2023.04.022)
Supplement: Supplementary file 1 — Supplementary material [file mmc1.pdf]

Supplementary Information

Spatiotemporal tracking of gold nanorods after intranasal administration for brain targeting

Shunping Han[1,6], Julie Tzu-Wen Wang[1], Emine Yavuz[1,5], Alaa Zam[1], Nadia Rouatbi[1], Rifka Nurul Utami[1], Revadee Liam-Or[1], Alexander Griffiths[2], Wayne Dickson[3,6], Jane Sosabowski[4] and Khuloud T. Al-Jamal[1,6]*

1. Institute of Pharmaceutical Science, Faculty of Life Sciences & Medicine, King's College London, Franklin-Wilkins Building, 150 Stamford Street, London SE1 9NH, United Kingdom
2. London Metallomics Facility, King's College London, Franklin-Wilkins Building, 150 Stamford Street, London SE1 9NH, United Kingdom
3. Department of Physics, King's College London, Strand, London WC2R 2LS, United Kingdom
4. Centre for Molecular Oncology, Barts Cancer Institute, Queen Mary University of London, Charterhouse Square, London EC1M 6BQ, United Kingdom
5. Advanced Technology Research and Application Center, Selcuk University, Aleaddin Keykubat Yerleskesi, Akademi Mah. Yeni Istanbul Cad. No: 355/C, Selcuklu, Konya, Turkey
6. London Centre for Nanotechnology, King's College London, Strand, London WC2R 2LS, United Kingdom

*Corresponding author: Khuloud T. Al-Jamal
E-mail address: khuloud.al-jamal@kcl.ac.uk

Keywords**:** Gold nanorods, intranasal administration, biodistribution, brain region-specific uptake, multi-modal imaging, glioma

Supplementary methods

Amine group detection by Ninhydrin assay.

Ninhydrin assay was used to detect the primary amine group in NH_2-PEG-SH. In brief, 100 µL of sample was mixed with 100 µL of Ninhydrin reagent (2% solution). The mixture was heated at 95 °C for 8 min in a water bath. After cooling down to room temperature, the mixture was diluted 20-fold with ethanol and the absorbance was read at 570 nm by a plate reader (BMG Labtech, USA). The standard curves of NH_2-PEG-SH was established in the concentrations between 0 – 1.5 mM using deionized water as solvent or 0 - 5 mM using DMSO as solvent for calculation.

PEG detection by iodine solution-based assay

The concentration of PEG was determined using iodine solution-based assay by the published method with modifications [1]. Iodine solution was prepared by dissolving 1.27 g iodine in 100 mL of 2% (w/v) potassium iodide. Samples were diluted with deionized water to final concentrations of PEG within the range of 0 - 20 µM. To an 800 µL of sample, 20 µL of iodine solution was added. The blank was prepared as above with deionized water instead of the sample. Solutions were agitated to ensure adequate mixing and then read at 535 nm by the plate reader. The standard curves of NH_2-PEG-SH with the concentrations between 0 - 20 µM was established and measured in the same condition for calculation.

DTPA detection by Gd^{3+}-Xylenol Orange assay

DTPA was detected using indirect Xylenol Orange assay by the method published previously with modifications [2]. Xylenol Orange tetrasodium salt (3 mg) was dissolved in acetic buffer (10 mL, pH 5.8) to obtain Xylenol Orange solution. The Xylenol Orange solution was prepared freshly before use. In brief, the synthesized DTPA-PEG-SH (100 µL) was mixed with excess $GdCl_3$ (100 µL) with known concentration and vortexed for 30 s. Afterwards, the above mixture (50 µL) was added with Xylenol Orange solution (500 µL). The free Gd^{3+} can coordinate with Xylenol Orange and develop violet color in acid condition. The concentration of free Gd^{3+} in the above mixture was measured by the plate reader which is proportional to the ratio of the absorbances at 573 and 433 nm. The standard curve of Gd^{3+} was established in the concentrations of 0 - 1 mM. DTPA concentration in the mixture was calculated by subtracting the free Gd^{3+} from the initial one.

Supplementary results

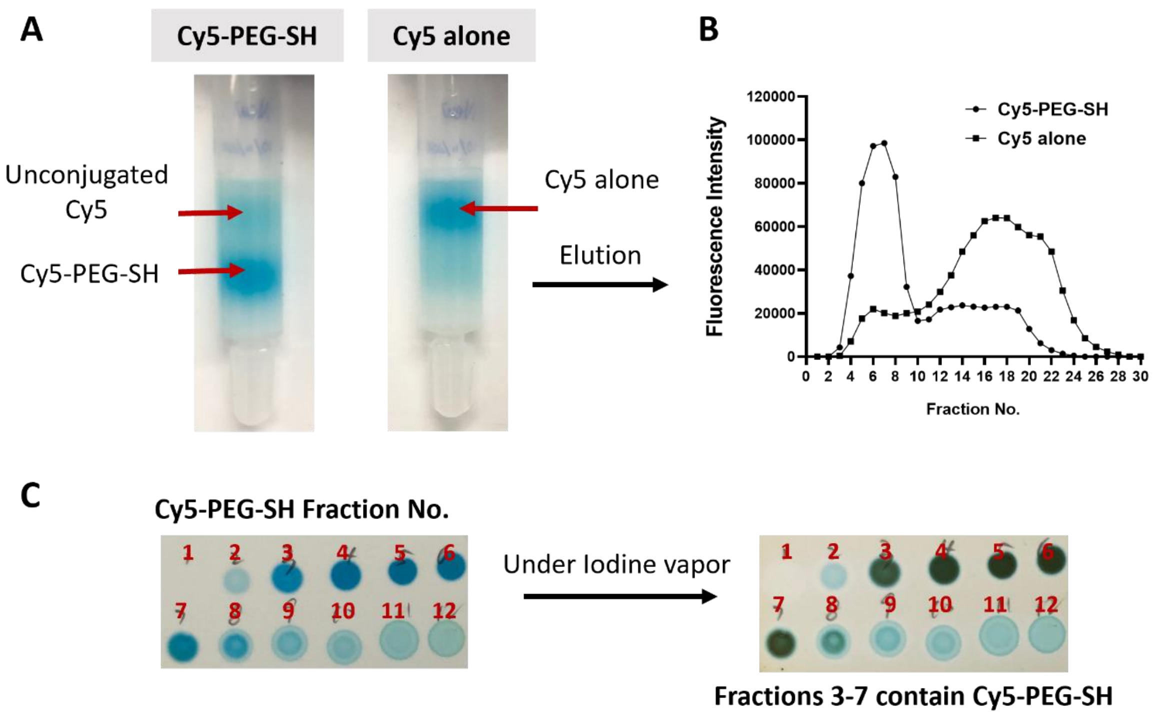

Figure S1. Confirmation of Cy5-PEG-SH linker synthesis. (A) After synthesis, Cy5-PEG-SH (500 µL) was added to a NAP™-5 column and entered the gel bed completely. Cy5 alone was applied as a control. **(B)** Samples were eluted by deionized water. Each fraction containing 120 µL of elution was collected and diluted 100-fold for fluorescence intensity measurement. Cy5-PEG-SH conjugates were eluted first followed by the unreacted sulfo-cyanine5 NHS ester. **(C)** A representative image of different Cy5-PEG-SH fractions on TLC plate. Only fractions which were both Cy5 and PEG fragment positive were collected.

Table S1. Optimization of NH_2-PEG-SH and DTPA anhydride conjugation.

NH_2-PEG-SH starting concentration (mM)[1]	DTPA anhydride starting concentration (mM)[1]	Molar ratio (PEG: DTPA anhydride)	Reaction time (h)	PEG substitution (%)[2-3]
2.5	5	1:2	4	81.2±12.0
	**2.5**	**1:1**		**75.4±18.6**
	1.25	2:1		48.9±3.3
	0.625	4:1		20.8±1.4
	5	1:2	24	76.4±18.3
	2.5	1:1		82.5±13.8
	1.25	2:1		51.5±1.3
	0.625	4:1		43.8±2.7

[1] Reaction volume is 0.5 mL using DMSO as the solvent.

[2] The substitution of amine groups in NH_2-PEG-SH was determined by Ninhydrin assay.

[3] PEG substitution is calculated by the following equation:

$$\text{PEG substitution (\%)} = \frac{\textit{Initial amine group concentration (mM)} - \textit{amine group concentration after reaction (mM)}}{\textit{Initial amine group concentration (mM)}}$$

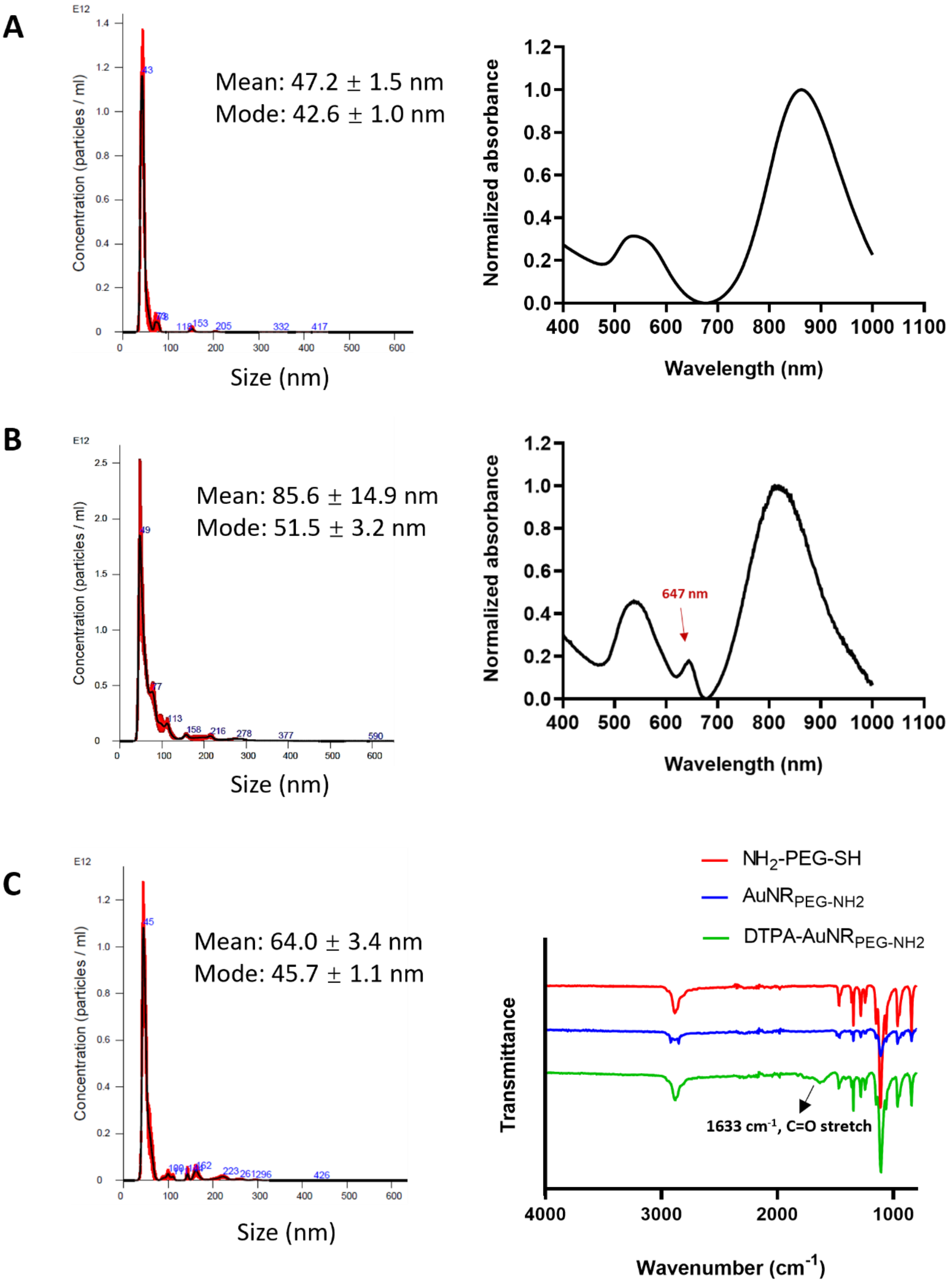

Figure S2. Characterization of AuNR$_{PEG-NH2}$, Cy5-AuNR$_{PEG-NH2}$ and DTPA-AuNR$_{PEG-NH2}$. (A) Representative hydrodynamic size distribution and UV–vis–NIR spectrum of AuNR$_{PEG-NH2}$. **(B)** Representative hydrodynamic size distribution and UV–vis–NIR spectrum of Cy5-AuNR$_{PEG-NH2}$. The conjugated Cy5-AuNR$_{PEG-NH2}$ demonstrated a typical peak which is consistent with the excitation wavelength of Cy5. **(C)** Representative hydrodynamic size distribution and FT-IR spectra of DTPA-AuNR$_{PEG-NH2}$. The typical PEG bonds and the amide bond at 1633 cm^{-1} in DTPA-AuNR$_{PEG-NH2}$ confirmed the successful DTPA-PEG-SH conjugation to AuNRs.

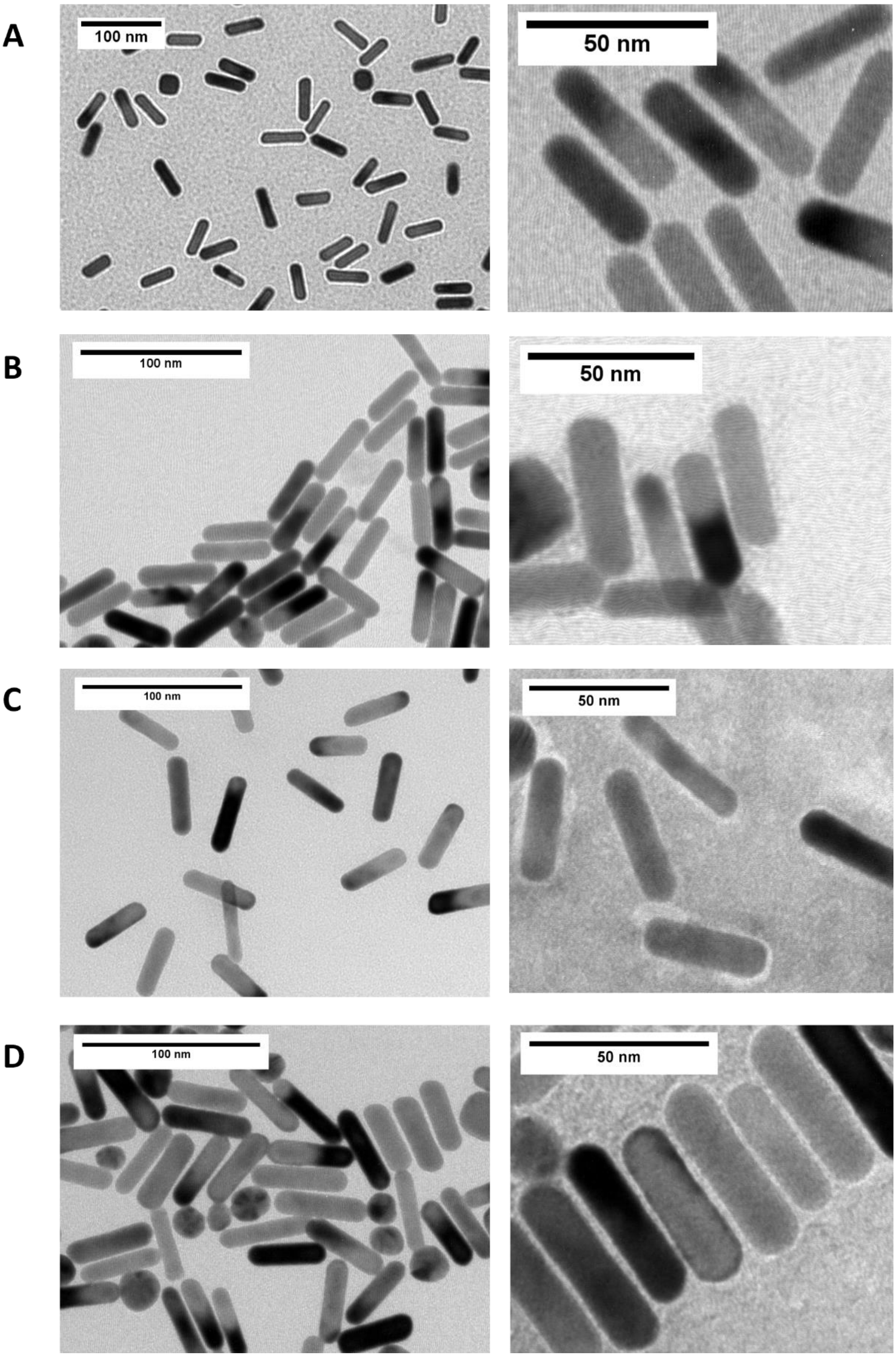

Figure S3. TEM images of (A) AuNR$_{CTAB,}$ (B) AuNR$_{PEG\text{-}NH2,}$ (C) Cy5-AuNR$_{PEG\text{-}NH2,}$ and (D) DTPA-AuNR$_{PEG\text{-}NH2}$ without (left) and with (right) uranyl acetate staining. For staining, samples were treated with 3% uranyl acetate for 2-3 min and then washed 2 times with filtered deionized water.

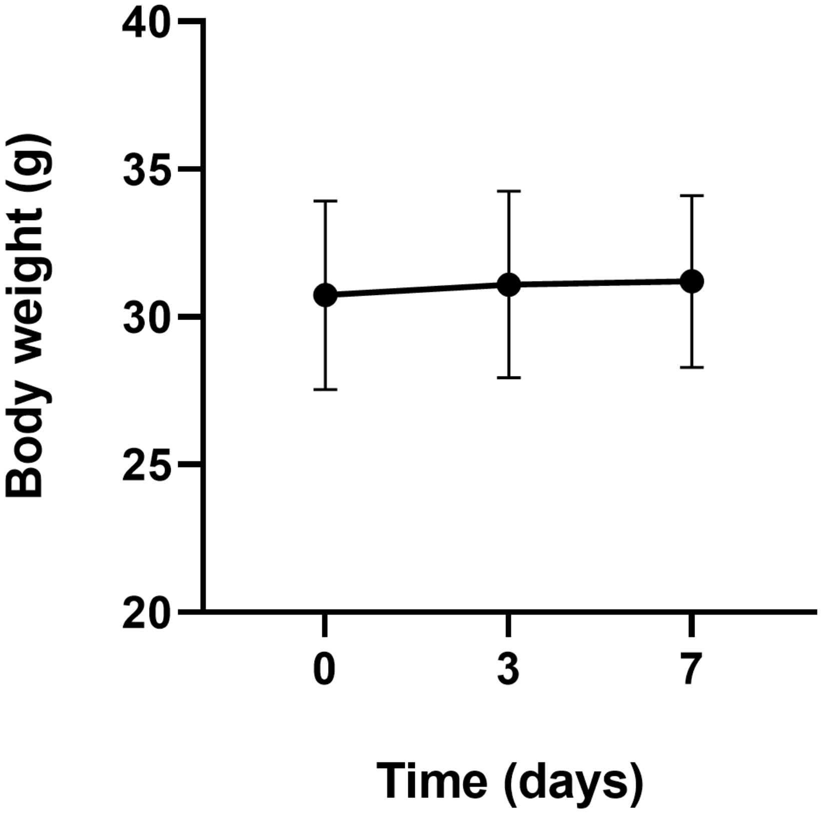

Figure S4. Whole body weight variation of CD-1 mice after intranasal administration of Cy5-AuNR$_{PEG\text{-}NH2}$ (20 µL, 300 nM of particles). Data are expressed as mean ± SD, n=3. There are no significant differences between different days, $P > 0.05$.

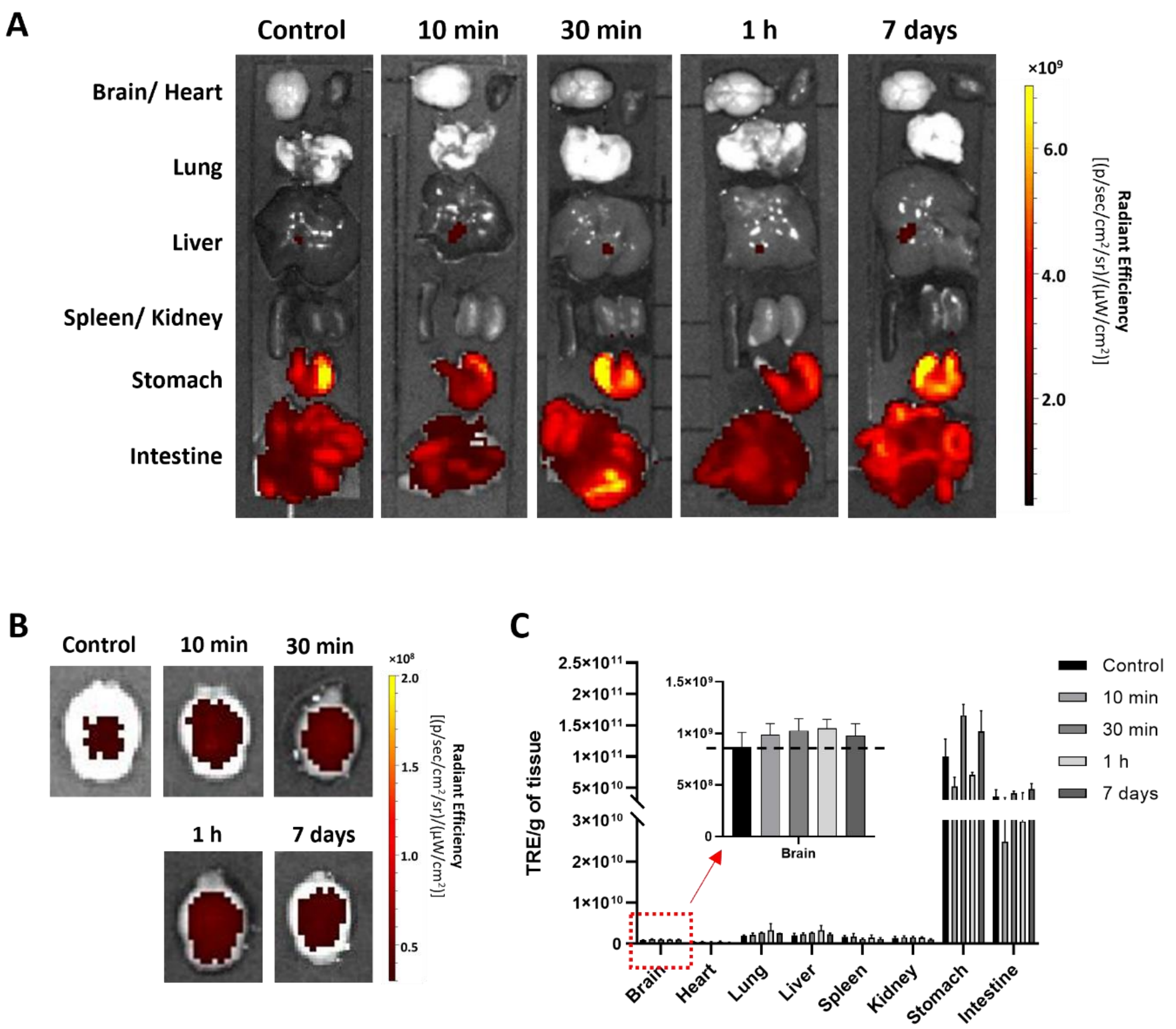

Figure S5. *Ex vivo* imaging of Cyanine5 in CD-1 mice after intranasal administration. Cyanine5 (20 µL) at equivalent fluorescence intensity to the formulations was intranasally administered into CD-1 mice. Representative *ex vivo* images of excised **(A)** organs and **(B)** brains harvested at 10 min, 30 min, 1 h and 7 days post-administration. Mice without any treatment were used as the control group. **(C)** *Ex vivo* quantification of fluorescence signals of Cyanine5 per gram of tissue at different time points. Values were expressed as mean ± SD, n=3. There are no significant differences in the brain fluorescence intensity between each group ($P > 0.05$). All images were obtained by IVIS Lumina® III and data were analysed by Living Image software.

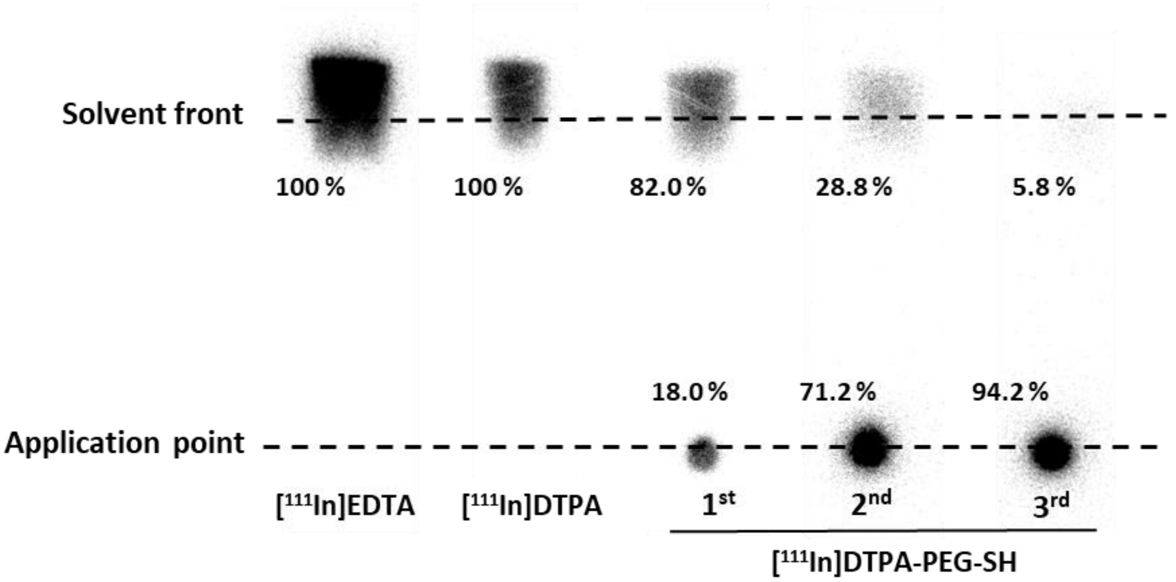

Figure S6. The Indium-111 complexation efficiency of DTPA-PEG-SH after running with PD-10 column with different times. The iTLC paper was run on 0.1 M ammonium acetate with 0.25 mM EDTA (pH 5.5) as the mobile phase and imaged using a phosphorimager. After running by PD-10 column with 3 rounds, the Indium-111 complexation efficiency was higher than 90%.

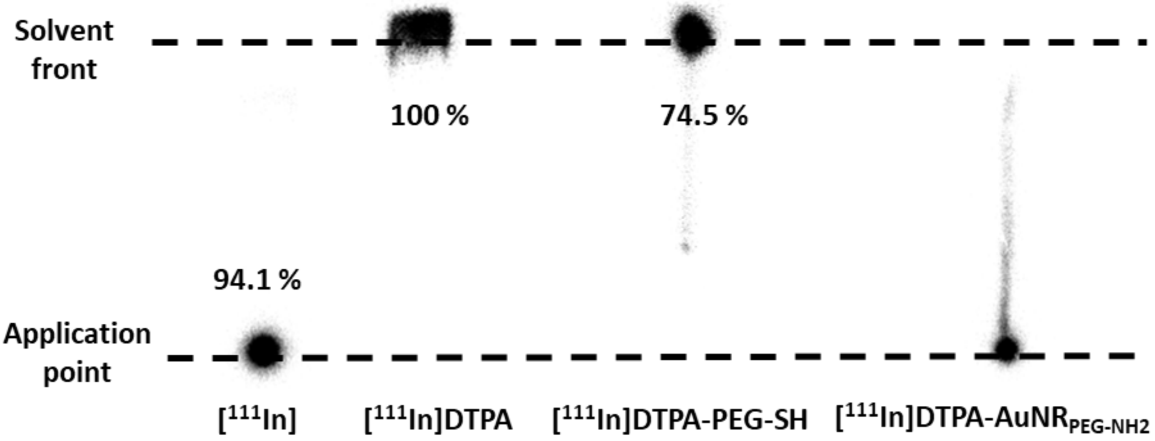

Figure S7. Contamination test of DTPA and DTPA-PEG-SH in DTPA-AuNR$_{PEG\text{-}NH2}$. The iTLC paper was run on 3.5% ammonia: methanol (1:1, v/v, pH 10.5) as the mobile phase and imaged using a phosphorimager. [^{111}In]DTPA and [^{111}In]DTPA-PEG-SH appeared as mobile spots at the solvent front. [^{111}In]DTPA-AuNR$_{PEG\text{-}NH2}$ demonstrated a migration from the application point without presenting significant radioactivity in the solvent front, confirming the high purity of [^{111}In]DTPA-AuNR$_{PEG\text{-}NH2}$.

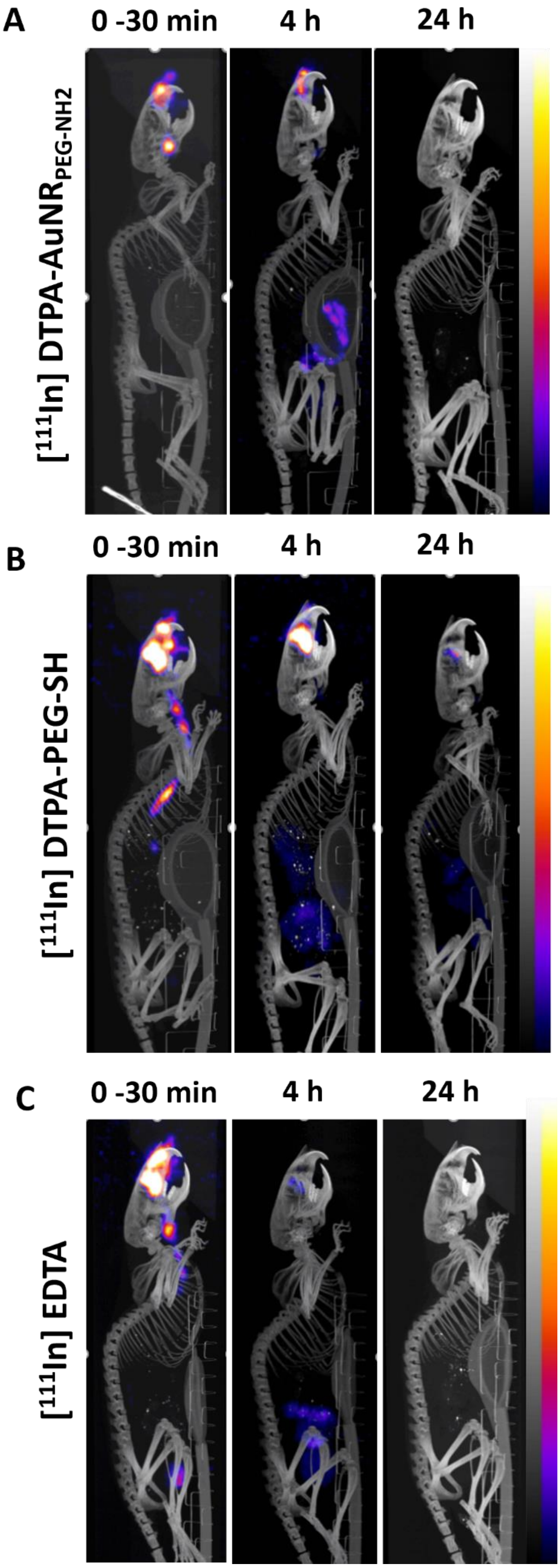
A
0 -30 min
4 h
24 h
[111In] DTPA-AuNRPEG-NH2
B
0 -30 min
4 h
24 h
[111In] DTPA-PEG-SH
C
0 -30 min
4 h
24 h
[111In] EDTA

125

Figure S8. Whole body SPECT/CT imaging of [^{111}In]-labelled (A) DTPA-AuNR$_{PEG\text{-}NH2}$, (B) DTPA-PEG-SH and (C) EDTA in CD-1 mice. CD-1 mice were intranasally administered with 5-10 MBq of [^{111}In]-labelled compounds. Imaging was done at 0-30 min, 4 h and 24 h post-administration.

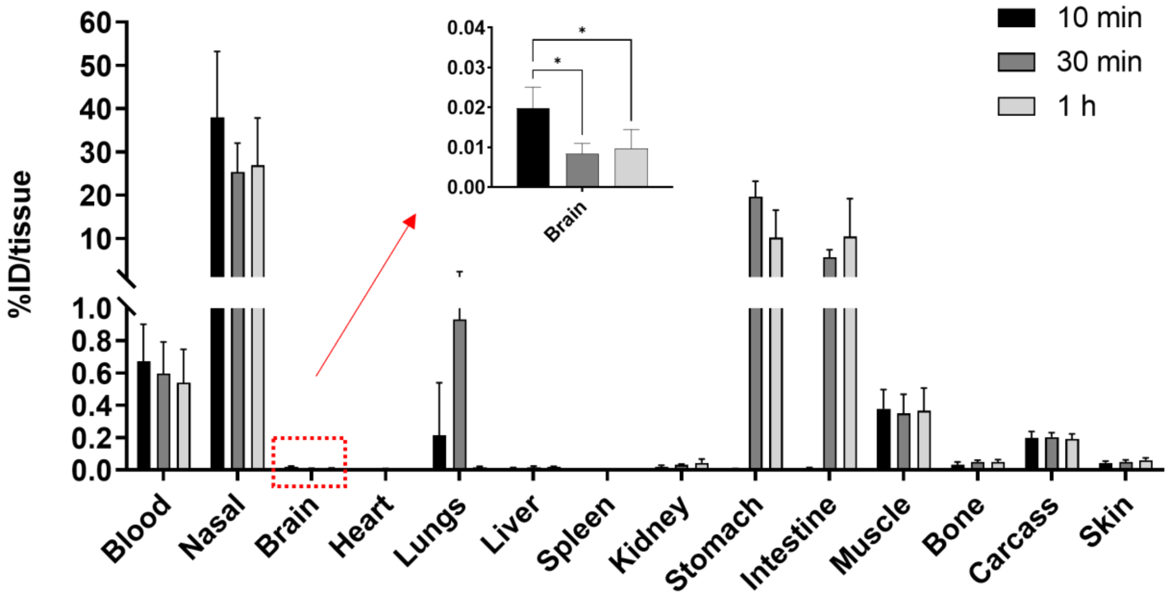

Figure S9. Organ biodistribution of [^{111}In]DTPA-AuNR$_{PEG\text{-}NH2}$. Animals were culled at 10 min, 30 min or 1 h post-administration. Inset shows the zoomed-in %ID per brain uptake.

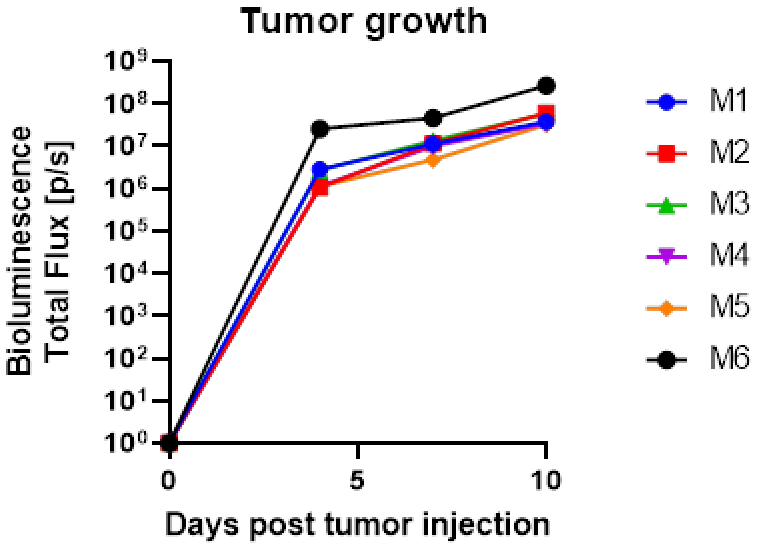

Figure S10. Tumor growth curves after GL261 cells injection. Murine GL261 cells (200 K, 2 µL) expressing Red-Fluc luciferase, were stereotactically inoculated into the left hemisphere of 4-6 weeks old C57BL/6 mice. Tumor

growth was monitored by whole-body Bioluminescence Imaging. Tumor growth was plotted as Bioluminescence intensity, expressed as photons/second (p/s) over time.

Reference

1. Gong, X.W., et al., *Discarded free PEG-based assay for obtaining the modification extent of pegylated proteins.* Talanta, 2007. **71**(1): p. 381-4.
2. Barge, A., et al., *How to determine free Gd and free ligand in solution of Gd chelates. A technical note.* Contrast media & molecular imaging, 2006. **1**(5): p. 184-188.
